# Enhanced diagnosis of axial spondyloarthritis using machine learning with sacroiliac joint MRI: a multicenter study

**DOI:** 10.1186/s13244-025-01967-x

**Published:** 2025-04-25

**Authors:** Zhuoyao Xie, Zefeiyun Chen, Qinmei Yang, Qiang Ye, Xin Li, Qiuxia Xie, Caolin Liu, Bomiao Lin, Xinai Han, Yi He, Xiaohong Wang, Wei Yang, Yinghua Zhao

**Affiliations:** 1 Department of Radiology, The Third Affiliated Hospital of Southern Medical University (Academy of Orthopedics Guangdong Province), Guangzhou, China; 2 Guangdong Provincial Key Laboratory of Medical Image Processing, School of Biomedical Engineering, Southern Medical University, Guangzhou, China; 3 Department of Radiology, The Sixth Affiliated Hospital of South China University of Technology, Nanhai, China; 4 Department of Radiology, Zhujiang Hospital of Southern Medical University, Guangzhou, China; 5 Department of Rheumatology and Immunology, The Third Affiliated Hospital of Southern Medical University (Academy of Orthopedics Guangdong Province), Guangzhou, China; 6 Department of Radiology, The Third Affiliated Hospital of Sun Yat-Sen University, Guangzhou, China

**Keywords:** Axial spondyloarthritis, Machine learning, Deep learning, MRI, Sacroiliac joints

## Abstract

**Objectives:**

To develop a machine learning (ML)-based model using MRI and clinical risk factors to enhance diagnostic accuracy for axial spondyloarthritis (axSpA).

**Methods:**

We retrospectively analyzed datasets from four centers (A–D), focusing on patients with chronic low back pain. A subset from center A was used for prospective validation. A deep learning (DL) model based on ResNet50 was constructed using sacroiliac joint MRI. Clinical variables were integrated with DL scores in ML algorithms to distinguish axSpA from non-axSpA patients. Model performance was assessed by the area under the receiver operating characteristic curve (AUC), sensitivity, specificity, and accuracy.

**Results:**

The study included 1294 patients (median age 31 years [interquartile range 24–42]; 35.5% females). Clinical risk factors identified were age, sex, and human leukocyte antigen-B27 status. The MRI-based DL model demonstrated an AUC of 0.837, 0.636, 0.724, 0.710, and 0.812 on the internal test set, three external test sets, and the prospective validation set, respectively. The combined model, particularly the K-nearest-neighbors-11 algorithm, demonstrated superior performance across multiple test sets with AUCs ranging from 0.853 to 0.912. It surpassed the Assessment of SpondyloArthritis International Society criteria with better AUC (0.858 vs. 0.650, *p* < 0.001), sensitivity (87.8% vs. 42.4%, *p* < 0.001), and accuracy (78.7% vs. 56.9%, *p* < 0.001).

**Conclusion:**

The ML method integrating MRI and clinical risk factors effectively identified axSpA, representing a promising tool for the diagnosis and management of axSpA.

**Clinical relevance statement:**

The machine learning model combining MRI and clinical risk factors potentially enables earlier diagnosis and intervention for axial spondyloarthritis patients, reducing the delays commonly associated with traditional diagnostic approaches.

**Key Points:**

- Axial spondyloarthritis (AxSpA) lacks definitive diagnostic criteria or markers, leading to diagnostic delay.
- MRI-based deep learning provided quantitative analysis of sacroiliac joint changes indicative of axSpA.
- A machine learning model combining sacroiliac joint MRI and clinical risk factors enhanced axSpA identification.

**Graphical Abstract:**

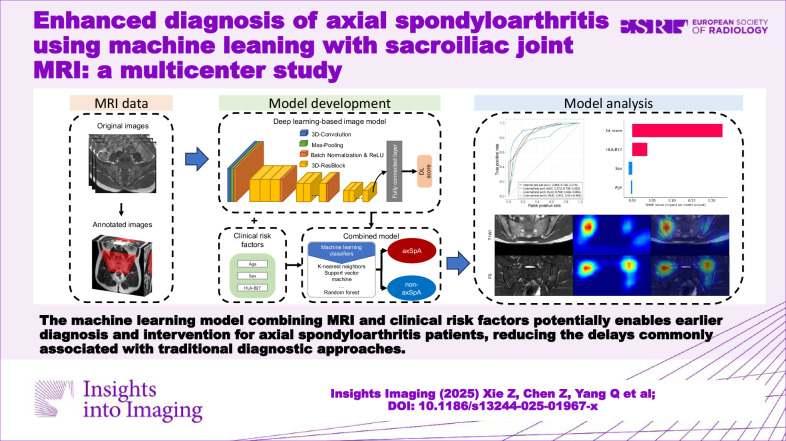

## Introduction

Axial spondyloarthritis (axSpA) is a chronic inflammatory disease that predominantly affects the sacroiliac joints (SIJs) in the early stages, with global prevalence ranging from 0.01 to 1.4% [1]. Patients commonly present with symptoms such as low back pain, stiffness, and progressive structural changes in the joints [2]. Without timely diagnosis and intervention, persistent inflammation may lead to bone erosion, sclerosis, and ankylosis, significantly increasing the risk of long-term disability [3]. Additionally, axSpA is associated with a higher incidence of comorbid conditions, such as hypertension, hyperlipidemia, and depression, further exacerbating the disease burden [4]. Given these risks, early and accurate diagnosis is crucial for initiating appropriate treatment strategies to alleviate symptoms, slow disease progression, and improve long-term outcomes.

Diagnosing AxSpA remains a significant challenge due to the absence of definitive biomarkers and the overlap of symptoms with other conditions [5, 6]. In clinical practice, the diagnosis is often guided by the Assessment of SpondyloArthritis International Society (ASAS) classification criteria, which emphasize key features such as inflammatory back pain (IBP), arthritis, and enthesitis [7]. MRI is crucial for the early detection of axSpA by providing detailed visualization of sacroiliitis and related changes, including bone marrow edema, erosion, and ankylosis [8]. However, MRI findings often overlap with other musculoskeletal disorders, such as degenerative arthritis, trauma, or infection, leading to diagnostic ambiguity and inconsistent interpretations [6]. These challenges are further exacerbated by a shortage of rheumatology specialists and the frequent misapplication of the ASAS criteria as a definitive diagnostic tool, resulting in a tendency to prioritize specificity over sensitivity, thereby delaying diagnosis by several years [5, 9].

In recent years, machine learning (ML) and deep learning (DL) have gained traction as innovative solutions for enhancing diagnostic accuracy in rheumatology [10]. ML algorithms have demonstrated success in leveraging structured clinical data (e.g., back pain, Crohn’s disease, and human leukocyte antigen [HLA]-B27) to identify axSpA [11]. Notably, DL models have shown promising performance in interpreting SIJ MRI, achieving accuracy comparable to expert radiologists in detecting axSpA-related changes [12, 13]. However, the potential of ML approaches integrating MRI data and clinical features for automated axSpA identification remains largely underexplored.

This study aimed to develop an ML model that combines SIJ MRI data and clinical risk factors to accurately identify axSpA. The model’s performance was also validated across multiple centers and compared against the ASAS classification criteria.

## Methods

### Ethics statement

This study received ethical approval from the Institutional Review Board of center A (No. 201501003) and additional approval from centers B, C, and D. Written informed consent was waived for retrospective data and obtained for prospective data, and all data were anonymized. The study adhered to the Standards for Reporting Diagnostic Accuracy (STARD) reporting guidelines [14] and the tenets of the Declaration of Helsinki [15].

### Study participants and datasets

**Fig. 1:**
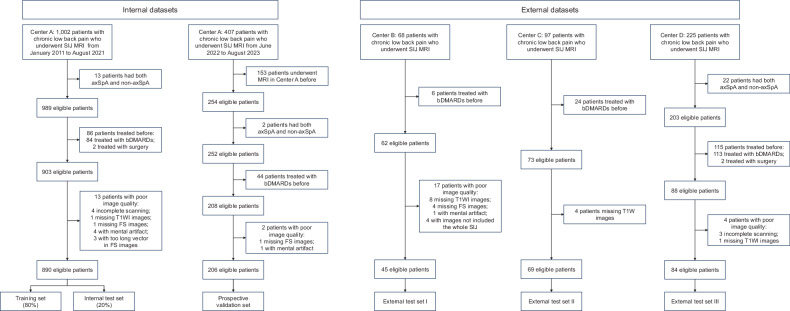
Flowchart of patient inclusion and exclusion. AxSpA, axial spondyloarthritis; SIJ, sacroiliac joint; bDMARDs, biologic disease-modifying anti-rheumatic drugs; T1WI, T1-weighted imaging; FS, fluid-sensitive fat suppression

Consecutive patients with chronic low back pain were recruited from four centers (centers A, B, C, and D) between January 2011 and July 2021 for retrospective data collection. Additionally, prospective data were gathered from Center A between August 2021 and August 2023. Inclusion and exclusion criteria are detailed in Supplementary Appendix S1. A flowchart of patient inclusion and exclusion is depicted in Fig. 1. Diagnostic assessment of axSpA and non-axSpA patients is detailed in Supplementary Appendix S2. Details regarding non-axSpA cases are demonstrated in Supplementary Appendix S3.

The retrospective dataset from center A was divided into training and internal test sets at a ratio of 5: 1. Data from centers B, C, and D were served as external test sets I, II, and III, respectively, while the prospective dataset from center A was used as the prospective validation set. The clinical data collected included demographic features (age and sex), disease duration, various spondyloarthritis (SpA) features (inflammatory back pain [IBP], arthritis, enthesitis, anterior uveitis, dactylitis, psoriasis, Crohn’s disease or ulcerative colitis, good response to nonsteroidal anti-inflammatory drugs [NSAIDs], SpA family history, HLA-B27 status, and C-reactive protein [CRP]), and erythrocyte sedimentation rate (ESR). The definitions of these SpA features are detailed in Table S1. All clinical data were extracted from electronic health records. For missing clinical data, multiple imputation methods were employed to estimate missing values, ensuring that the complete datasets were used in subsequent analyses.

### MRI protocols

All patients underwent SIJ MRI in the supine position, with scans acquired in both oblique axial and oblique coronal planes. MRI examinations were conducted using 1.5-T and 3.0-T scanners from various manufacturers. The three MRI sequences included T1-weighted imaging (T1WI), T2-weighted imaging (T2WI), and fluid-sensitive fat suppression (FS) sequences. The MRI protocols are detailed in Table S2.

### Image analysis and image preprocessing

Eligible MRI images were downloaded from the picture archiving and communication system (PACS) in their original resolution and stored as Digital Imaging and Communications in Medicine (DICOM) files. These images were randomly distributed to three musculoskeletal radiologists (Q.X.X., X.L., and Q.M.Y.; with 4, 5, and 5-year experience, respectively), who were blinded to clinical information and final diagnosis. They independently delineated bounding boxes using ITK-SNAP software (version 4.0.2), encompassing the ilium, sacrum and bilateral SIJs. The bounding boxes were standardized based on the largest slice of the iliac bone and extended uniformly across all SIJ slices to form a 3D bounding box. A senior radiologist (Y.H.Z.; with 34-year experience) reviewed all bounding boxes, and any discrepancies were resolved by consensus. Image preprocessing is detailed in the Supplementary Appendix S4.

### MRI-based deep learning model development

**Fig. 2:**
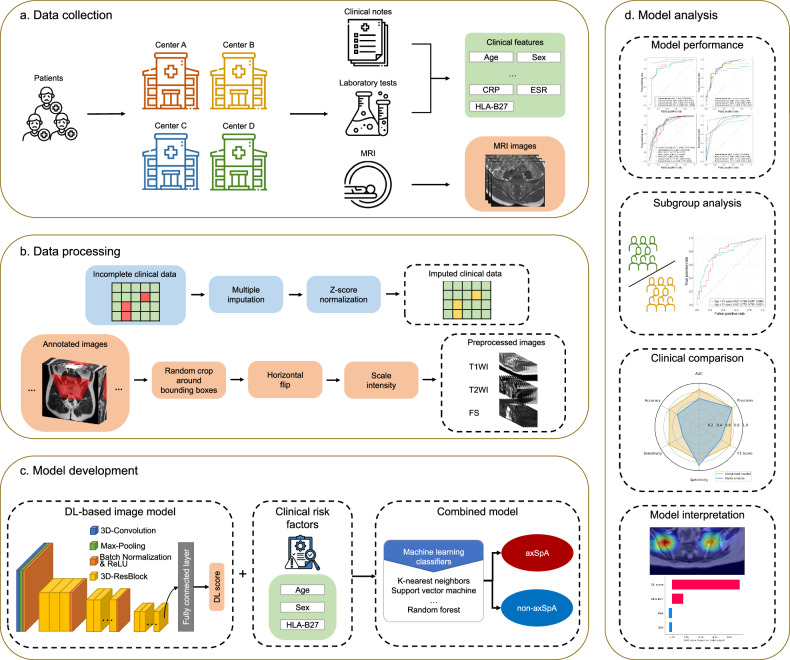
Study design framework. **a** MRI and clinical data of patients were collected from four centers, focusing on patients with chronic low back pain. **b** Clinical data underwent imputation for missing features, while sacroiliac joints on MRI images were manually annotated with bounding boxes for subsequent analysis. **c** MRI-based DL models were constructed using a 3D ResNet50 architecture. In parallel, ML models were constructed based on different algorithms integrating the DL score and clinical risk factors. **d** The performance of the constructed models was evaluated across all centers. The diagnostic accuracy of the ML models was compared to the current ASAS classification criteria. In addition, model interpretability was enhanced by visualization techniques. ASAS, the Assessment of SpondyloArthritis International Society; DL, deep learning; ML, machine learning; CRP, C-reactive protein; ESR, erythrocyte sedimentation rate; HLA, human leukocyte antigen; ReLU, rectified linear unit

We used the ResNet-50 [16] architecture as the backbone as the backbone for our DL models due to its exceptional performance and robustness in medical image analysis across diverse datasets. This approach involved building three separate DL models based on different MRI sequences (T1WI, T2WI, and FS), given the variations in acquisition parameters. Standard 2D convolutional and pooling layers in ResNet blocks were replaced with their 3D counterparts to accommodate the volumetric nature of MRI data (Fig. 2c). Each DL model produced a prediction score reflecting the likelihood of axSpA based on the respective MRI sequence. To enhance predictive performance, we constructed DL models that integrated outputs from the single-sequence DL models: three two-sequence models (T1WI + T2WI, T1WI + FS, T2WI + FS) and one three-sequence model (T1WI + T2WI + FS). The prediction scores from the single-sequence models were aggregated using summation and averaging methods to produce final predictions for the multi-sequence models. Subgroup analyses were performed to evaluate the performance of the MRI-based models across all test sets. Further details on the training and selection processes of the models are provided in Supplementary Appendix S5–S7.

### Machine learning combined model development

Initially, univariate logistic regression analysis was employed to investigate six clinical variables: age, sex, disease duration, CRP, ESR, and HLA-B27, to identify potential clinical indicators. Subsequently, multivariate analysis was conducted to determine independent clinical risk factors from the significant variables identified in the univariate analysis. These independent clinical risk factors, along with the DL score derived from the MRI-based model, were utilized to develop combined models using seven ML algorithms: logistic regression, Naïve Bayes, K-nearest-neighbors (KNN, with K = 5, 7, 9, and 11), support vector machine, decision tree, and random forest. The experimental environment is detailed in Supplementary Appendix S8. All code is available at https://github.com/SMU-MedicalVision/CEAS.

### Model interpretation

For model interpretation, we applied two advanced techniques: gradient-weighted class activation mapping (Grad-CAM) [17] for the MRI-based DL model and Shapley additive explanation (SHAP) [18] for the machine learning combined model. Details of model interpretation are described in Supplementary Appendix S9.

### Evaluation using the ASAS classification criteria

Patients were evaluated as axSpA or non-axSpA using the ASAS classification criteria, including the image arm and the clinical arm (Fig. S1) [7]. Each patient across all test sets was evaluated based on these criteria. Specifically, the ASAS criteria item, ‘sacroiliitis on imaging’, was determined by consulting the corresponding radiology report. Additionally, SpA features were obtained from the electronic medical record system.

### Statistical analysis

Statistical analyses were conducted using R statistical software (version 4.3.3; R Core Team) and Python (version 3.10.10; Python Software Foundation). Comparisons were made using the Mann–Whitney U test and the Kolmogorov–Smirnov test for continuous variables, and the chi-square test for categorical variables. Spearman correlation analysis was performed to evaluate the correlation between variables. Model performance was evaluated using several metrics, including the area under the receiver operating characteristic curve (AUC), accuracy, sensitivity, specificity, F1 score, and precision. We calculated 95% confidence intervals for all metrics using 1000 bootstrap samples. AUCs were compared using the Delong test, while accuracy, sensitivity, and specificity were compared using the McNemar test. A two-sided *p*-value less than 0.05 indicated statistical significance.

## Results

### Patient characteristics and datasets

A total of 1294 patients (age, 31 [24–42] years; 459 [35.5%] females) were analyzed (Table 1). The three most prevalent non-axSpA diagnoses were nonspecific low back pain (148 [37.2%]), degenerative arthritis (42 [10.6%]), and undifferentiated arthritis (38 [9.5%]). The external test sets I and III included older patients (*p* < 0.001), while the external test set III and prospective validation set had a higher proportion of females (*p* < 0.001). Notably, most patients in the external test set I and prospective validation set were HLA-B27-negative (25 [55.6%] and 199 [48.1%], respectively). The external test set II exhibited the highest percentage of patients with unknown HLA-B27 status (31 [44.9%]) (Fig. S2). There were no statistically significant differences between the clinical data from multiple imputation and the original data for ESR and CRP (Fig. S3).

**Table 1:** Patient characteristics

Patient characteristics	Overall (1294)	Training set (712)	Internal test set (178)	External test set I (45)	External test set II (69)	External test set III (84)	Prospective validation set (206)	*p*-value
Age (years)	31 (24–42)	30 (24–40)	29 (23.2–42.5)	35 (30–44)	32 (23–44)	35 (28–43)	30 (25–44)	< 0.001***
Sex								< 0.001***
Female	459 (35.5%)	247 (34.7%)	57 (32.0%)	14 (31.1%)	13 (18.8%)	32 (38.1%)	96 (46.6%)	
Male	835 (64.5%)	465 (65.3%)	121 (68.0%)	31 (68.9%)	56 (81.2%)	52 (61.9%)	110 (53.4%)	
Disease								0.377
AxSpA	896 (69.2%)	489 (68.7%)	122 (68.5%)	26 (57.8%)	50 (72.5%)	57 (67.9%)	152 (73.8%)	
Non-axSpA	398 (30.8%)	223 (31.3%)	56 (31.5%)	19 (42.2%)	19 (27.5%)	27 (32.1%)	54 (26.2%)	
Disease duration (months)	24 (6–60)	24 (7–60)	24 (6–69)	11 (5–36)	24 (6–96)	60 (12–120)	12 (3–48)	< 0.001***
ESR (mm/h)	15 (7–34)	15 (7–31)	15 (7–30)	19 (9–34)	42.5 (23–62.5)	26 (7.2–52)	13.5 (6–25)	< 0.001***
Missing data	128 (9.9%)	61 (8.6%)	14 (7.9%)	6 (13.3%)	21 (30.4%)	6 (7.1%)	20 (9.7%)	
CRP (mg/L)	4.6 (1.0–16.4)	4.1 (0.8–14.4)	5.0 (1.0–14.0)	6.3 (2.4–11.7)	21.7 (8.9–46.8)	6.1 (1.1–24)	2.3 (0.9–12.6)	< 0.001***
Missing data	128 (9.9%)	54 (7.6%)	16 (9.0%)	8 (17.8%)	19 (27.5%)	11 (13.1%)	18 (8.7%)	
HLA-B27								< 0.001***
Positive	584 (45.1%)	312 (43.8%)	82 (46.1%)	14 (31.1%)	33 (47.8%)	57 (67.9%)	86 (41.7%)	
Negative	492 (38.0%)	277 (38.9%)	67 (37.6%)	25 (55.6%)	5 (7.2%)	19 (22.6%)	99 (48.1%)	
Missing data	218 (16.8%)	123 (17.3%)	29 (16.3%)	6 (13.3%)	31 (44.9%)	8 (9.5%)	21 (10.2%)	
Inflammatory back pain	754 (58.3%)	431 (60.5%)	111 (62.4%)	21 (46.7%)	38 (55.1%)	31 (36.9%)	122 (59.2%)	< 0.001***
Arthritis	526 (40.6%)	347 (48.7%)	71 (39.9%)	33 (73.3%)	6 (8.7%)	35 (41.7%)	34 (16.5%)	< 0.001***
Enthesitis (heel)	58 (4.5%)	21 (2.9%)	4 (2.2%)	26 (57.8%)	0 (0.0%)	4 (4.8%)	3 (1.5%)	< 0.001***
Uveitis	29 (2.2%)	22 (3.1%)	4 (2.2%)	0 (0.0%)	0 (0.0%)	2 (2.4%)	1 (0.5%)	0.188
Dactylitis	94 (7.3%)	50 (7.0%)	10 (5.6%)	10 (22.2%)	1 (1.4%)	12 (14.3%)	11 (5.3%)	< 0.001***
Crohn’s/colitis	3 (0.2%)	0 (0.0%)	1 (0.6%)	1 (2.2%)	0 (0.0%)	0 (0.0%)	1 (0.5%)	0.053
Psoriasis	11 (1.0%)	3 (0.4%)	2 (1.1%)	0 (0.0%)	1 (1.3%)	4 (5.3%)	1 (0.4%)	0.013*
Good response to NSAID								< 0.001***
Yes	251 (19.4%)	130 (18.3%)	30 (16.9%)	12 (26.7%)	29 (42.0%)	29 (34.5%)	21 (10.2%)	
No	236 (18.2%)	108 (15.2%)	25 (14.0%)	13 (28.9%)	4 (5.8%)	12 (14.3%)	74 (35.9%)	
Missing data	807 (62.4%)	474 (66.6%)	123 (69.1%)	20 (44.4%)	36 (52.2%)	43 (51.2%)	111 (53.9%)	
Family history of SpA	63 (4.9%)	30 (4.2%)	4 (2.2%)	8 (17.8%)	3 (4.3%)	8 (9.5%)	10 (4.9%)	0.001**

Data are presented as number (%) or median (interquartile)

The Kruskal–Wallis test was used for continuous variables and the Chi-square test or Fisher’s exact test for categorical variables

Missing data for continuous variables were not used for statistical comparisons

*AxSpA* axial spondyloarthritis, *ESR* erythrocyte sedimentation rate, *CRP* C-reactive protein, *HLA* human leukocyte antigen, *NSAID* nonsteroidal anti-inflammatory drug, *SpA* spondyloarthritis

* *p* < 0.05; ** *p* < 0.01; *** *p* < 0.001

### Performance of MRI-based deep learning models

**Fig. 3:**
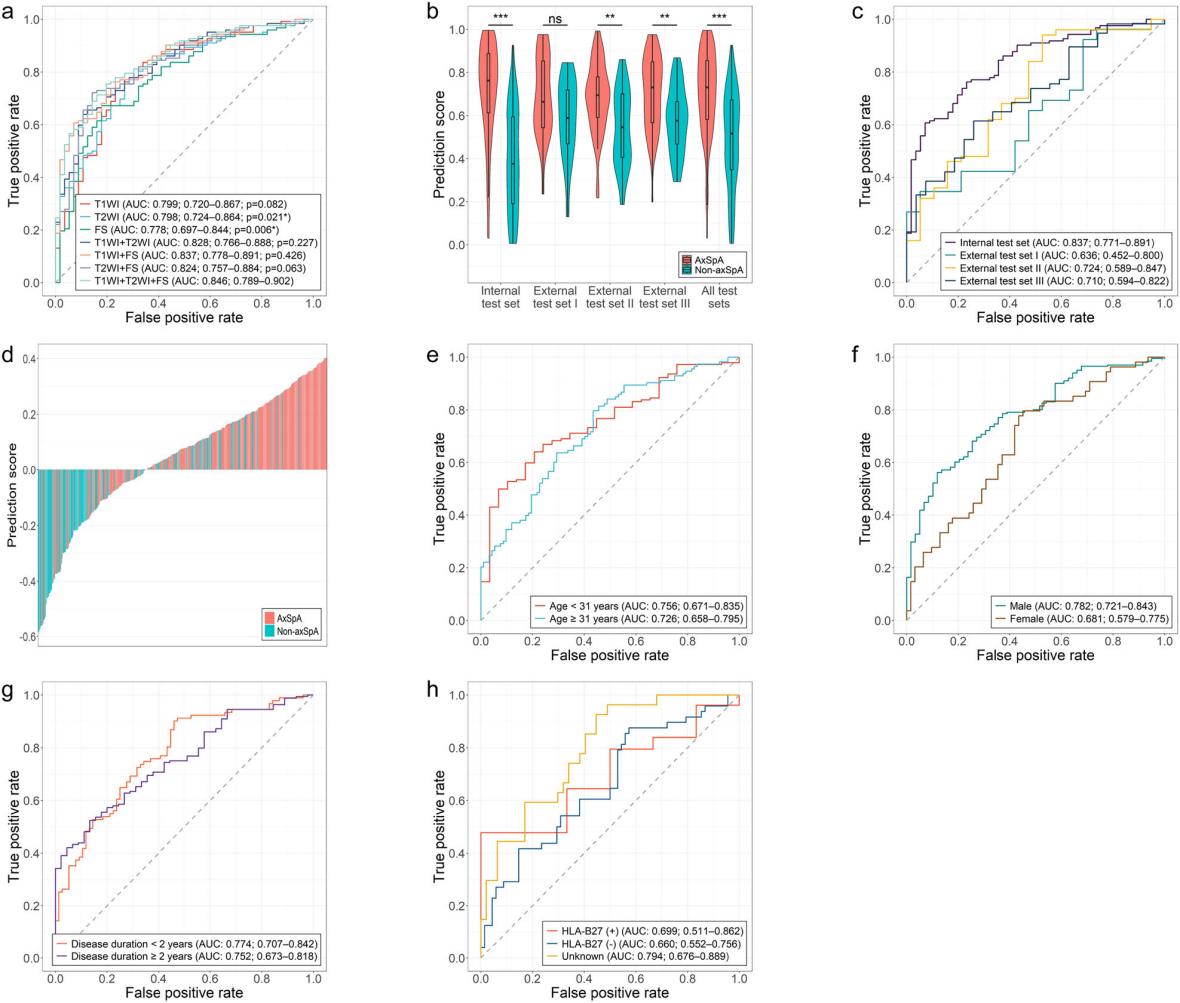
Performance of MRI-based DL models for classifying axSpA and non-axSpA. **a** ROC curves of DL model using single- and multi-sequence MRI on the internal set. **b** The violin plot demonstrating the distribution of the DL score based on ResNet50 across all test sets for axSpA versus non-axSpA groups. **c** ROC curves of the DL model based on T1WI + FS sequences across all test sets. **d**–**g** ROC curves of the DL model based on T1WI + FS sequences for subgroup analysis across all test sets, including age (**d**), sex (**e**), disease duration (**f**), and HLA-B27 status (**g**). **h** Waterfall plot illustrating DL score distribution across all test sets. AxSpA, axial spondyloarthritis; AUC, area under the receiver operating characteristic curve; HLA, human leukocyte antigen; ROC, receiver operating characteristic; T1WI, T1-weighted imaging; T2WI, T2-weighted imaging; FS, fluid-sensitive fat suppression; DL, deep learning. * *p* < 0.05

Among the single- and dual-sequence MRI-based DL models, the T1WI + FS model exhibited the highest AUC of 0.837 (95% CI: 0.778–0.891) on the internal test set (Fig. 3a; Table S3). Notably, there was no statistical difference between the performance of the T1WI + FS and T1WI + T2WI + FS models (*p* > 0.05; Fig. 3a). Due to the good performance of the T1WI + FS model and the fact that T1WI and FS are the sequences recommended by the ASAS criteria, we selected the T1WI + FS model as the final MRI-based model. The model demonstrated significantly higher DL scores in axSpA patients compared to non-axSpA patients across all test sets (Fig. 3b). The model achieved AUCs of 0.636 (95% CI: 0.452–0.800), 0.724 (95% CI: 0.589–0.847), and 0.710 (95% CI: 0.594–0.822) on the three external test sets (sets I, II, and III, respectively) (Fig. 3c), demonstrating consistent performance across diverse datasets. By adjusting the cutoff (Supplementary Appendix S10; Table S4), the MRI-based model achieved 76.4% accuracy, 77.0% sensitivity, and 71.4% specificity on the internal test set. For the external test sets I, II, and III, the model achieved accuracy ranging from 60.0 to 68.1%, sensitivity between 65.4% and 74.0%, and specificity from 20.9 to 52.6% (Table 2). The waterfall plot illustrated the ascending distribution of DL prediction scores, revealing that scores above the best cutoff predominantly corresponded to axSpA patients (Fig. 3d). The MRI-based model demonstrated comparable performance across age, sex, disease duration, and HLA-B27 subgroups (Fig. 3e–h; Table S5).

**Table 2:** Performance of the MRI-based deep learning model and the KNN-11-based combined model

Model	AUC	Accuracy	Sensitivity	Specificity	F1 score	Precision
MRI-based DL model						
Internal test set	0.837 (0.776–0.896)	76.4% (70.2–82.0%)	77.0% (69.7–84.0%)	75.0% (63.3–86.4%)	81.7% (75.4–86.7%)	87.0% (80.4–93.3%)
External test set I	0.636 (0.470–0.793)	60.0% (46.7–75.6%)	65.4% (48.0–84.0%)	52.6% (30.0–73.7%)	65.4% (48.8–78.7%)	65.4% (45.8–84.6%)
External test set II	0.724 (0.578–0.850)	68.1% (56.5–79.7%)	74.0% (61.4–85.7%)	52.6% (28.6–75.0%)	77.1% (66.7–85.4%)	80.4% (68.4–91.1%)
External test set III	0.710 (0.589–0.821)	66.7% (56.0–76.2%)	73.7% (62.3–84.7%)	51.9% (32.3–70.4%)	75.0% (64.8–83.2%)	76.4% (65.5–87.0%)
KNN-11-based combined model						
Internal test set	0.853 (0.792–0.910)	80.3% (74.7–86.0%)	82.8% (75.4–89.3%)	75.0% (62.3–86.0%)	85.2% (80.2–89.8%)	87.8% (81.4–93.7%)
External test set I	0.872 (0.768–0.953)	80.0% (68.9–91.1%)	92.3% (81.5–100.0%)	63.2% (40.0–84.2%)	84.2% (73.5–93.1%)	77.4% (62.1–91.7%)
External test set II	0.780 (0.658–0.895)	73.9% (63.8–84.1%)	80.0% (68.1–90.9%)	57.9% (34.7–81.0%)	81.6% (71.9–88.9%)	83.3% (72.1–93.5%)
External test set III	0.912 (0.841–0.966)	86.9% (79.8–94.0%)	89.5% (81.4–96.6%)	81.5% (66.7–95.5%)	90.3% (83.9–95.6%)	91.1% (83.3–98.1%)

*AUC* area under the receiver operating characteristic curve

### Performance of machine learning combined models

**Fig. 4:**
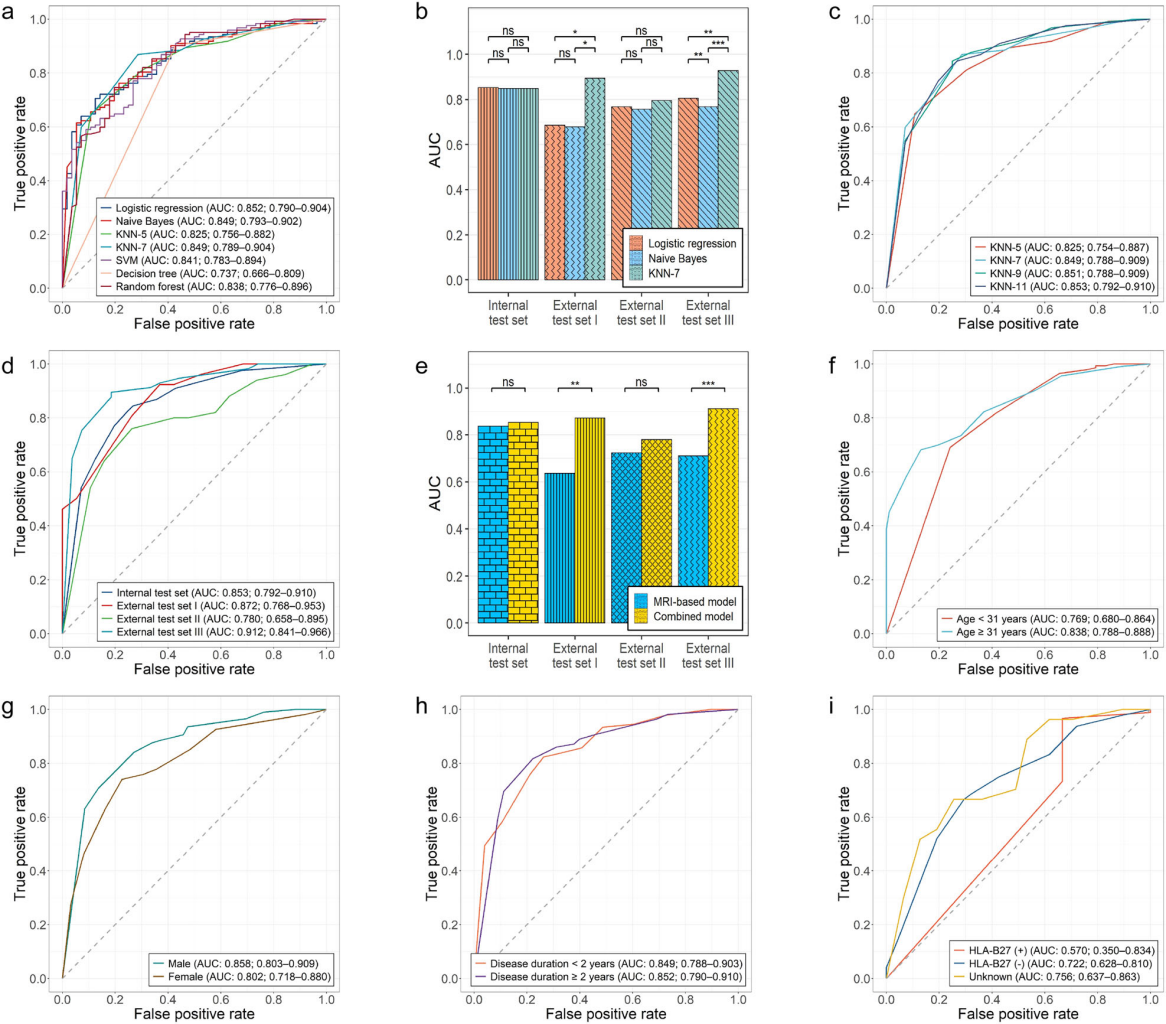
Performance of the combined model for classifying axSpA and non-axSpA. **a** ROC curves of the combined model based on different machine learning algorithms on the internal test set. **b** Bar plot for AUCs of the top 3 machine learning combined models on the internal and external test sets. **c** ROC curves of the KNN model varying K values on the internal test sets. **d** ROC curves of the KNN model with varying K values on the internal and external test sets. **e**–**h** ROC curves of the combined model for subgroup analysis across all test sets, including age (**e**), sex (**f**), disease duration (**g**), and HLA-B27 status (**h**). **i** Bar plot for AUCs of the imaging and combined models. AUC, area under the receiver operating characteristic curve; HLA, human leukocyte antigen; ROC, receiver operating characteristic; KNN, K-nearest-neighbors; SVM, support vector machine. * *p* < 0.05; ** *p* < 0.01; *** *p* < 0.001

Univariate and multivariate analyses identified age, sex, and HLA-B27 status as independent clinical risk factors for distinguishing axSpA from non-axSpA (Table S6). Among the top-performing ML combined models on the internal test set, logistic regression, Naïve Bayes, and KNN-7 models stood out (Fig. 4a; Table S7). Notably, the KNN-7-based model outperformed both logistic regression and Naïve Bayes on the external test sets I and III (Fig. 4b). Further exploration of different K values revealed that the KNN-11 model delivered the highest AUC of 0.853 (95% CI: 0.792–0.910) among all KNN-based models on the internal test set (Fig. 4c; Table S8) and was selected for further analysis. The model achieved an AUC of 0.872 (95% CI: 0.768–0.953), 0.780 (95% CI: 0.658–0.895), and 0.912 (95% CI: 0.841–0.966) on the external test sets I, II, and III, respectively (Fig. 4d). Using an optimal cutoff of 0.637 based on the highest Youden index, the model demonstrated adequate specificity (57.9–81.5%) and sensitivity (80.0–92.3%) across the external test sets (Tables 2 and S9). Notably, the KNN-11-based combined model outperformed the MRI-based model, yielding higher AUCs on the external test sets I and III (Fig. 4e). The combined model showed reliable performance across subgroups stratified analysis by age (Fig. 4f; AUCs of 0.769–0.838), sex (Fig. 4g; AUCs of 0.802–0.858), and disease duration (Fig. 4h; AUCs of 0.849–0.852) (Table S10). Moreover, it exhibited good performance for patients with HLA-B27-negative and unknown HLA-B27 status (Fig. 4i; AUC of 0.756). However, its performance was relatively weaker in the HLA-B27-positive subgroup (AUC of 0.570).

### Prospective validation

Refinement of the MRI-based and combined models using data from 61 patients during the first 3 months of the prospective validation set showed improved performance. The refined MRI-based model (AUC, 0.812 vs. 0.797, *p* > 0.05) and the combined model (AUC, 0.840 vs. 0.832, *p* > 0.05) outperformed their non-refined counterparts (Table S11).

### Comparison between the combined model and the ASAS classification criteria

**Fig. 5:**
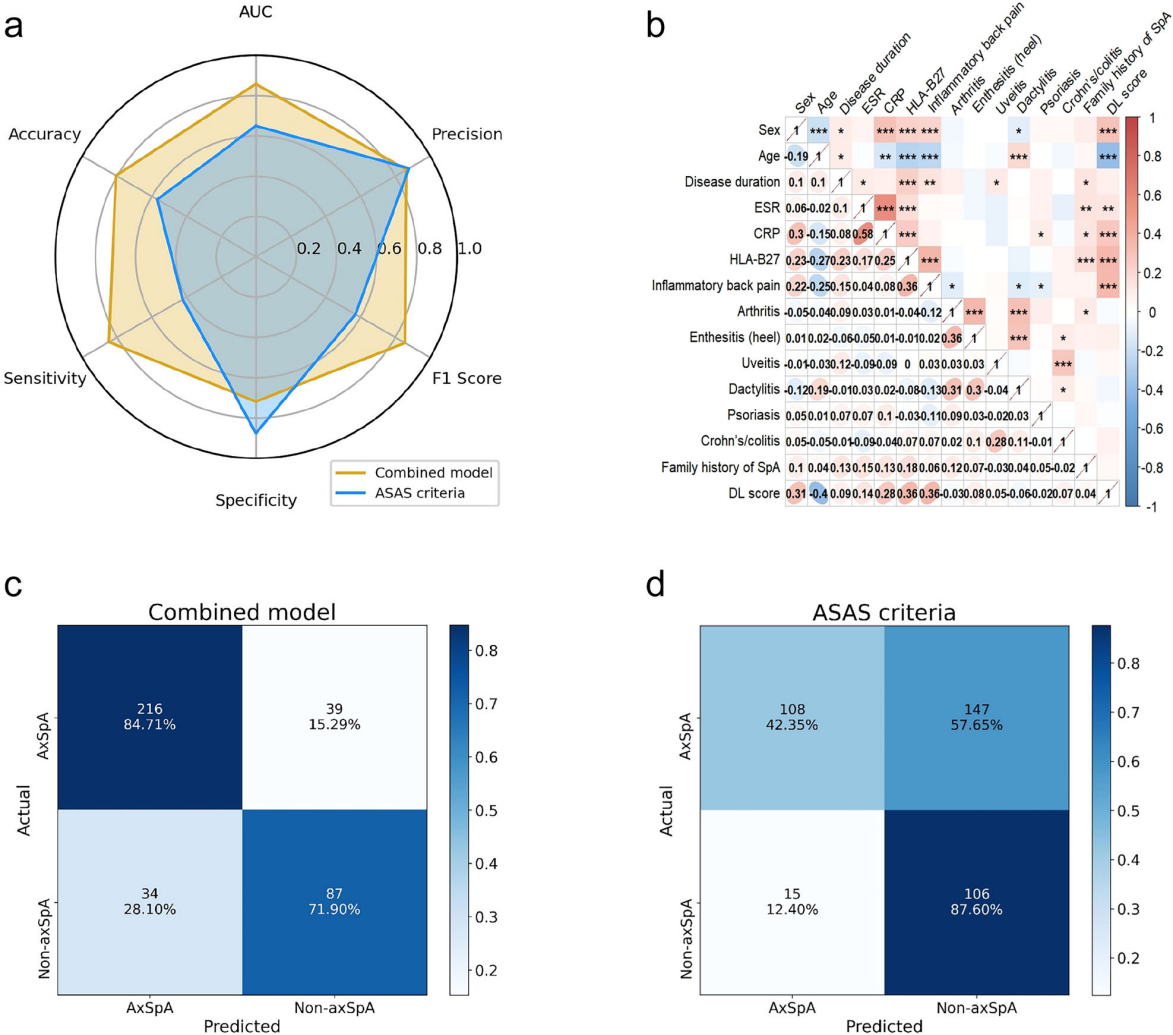
Performance comparison between the combined model and the ASAS classification criteria. **a** Radar plot comparing performance metrics of the combined model and the ASAS classification criteria across multiple metrics. **b** Correlation heatmap of variables across all test sets. **c** Confusion matrix of the combined model. **d** Confusion matrix of the ASAS classification criteria. ASAS, the Assessment of SpondyloArthritis International Society; AUC, area under the receiver operating characteristic curve. * *p* < 0.05; ** *p* < 0.01; *** *p* < 0.001

The radar chart illustrates that the combined model outperforms the ASAS criteria with higher AUC (0.858 vs. 0.650, *p* < 0.001), accuracy (78.7% vs. 56.9%, *p* < 0.001), sensitivity (87.8% vs. 42.4%, *p* < 0.001), and F1 score (84.8% vs. 57.1%) (Fig. 5a; Table 3). However, the model’s specificity was lower than that of the ASAS criteria. Significant correlations were observed between the DL score and age, sex, ESR, CRP, HLA-B27 status, and IBP (Fig. 5b). The confusion matrix indicates that the combined model more accurately identifies axSpA (Fig. 5c), whereas the ASAS criteria are more likely to classify patients as non-axSpA (Fig. 5d).

**Table 3:** Performance comparison between the combined model with the ASAS classification criteria across all test sets

	AUC	Accuracy	Sensitivity	Specificity	F1 score	Precision
Combined model	0.858 (0.817–0.896)	78.7% (74.7–83.0%)	87.8% (83.6–91.7%)	59.5% (50.8–67.8%)	84.8% (81.5–88.2%)	82.1% (77.7–86.6%)
ASAS criteria	0.650 (0.585–0.645)	56.9% (43.3–54.0%)	42.4% (19.5–30.6%)	87.6% (95.8–100.0%)	57.1% (32.5–46.6%)	87.8% (92.4–100.0%)
*p*-value	< 0.001***	< 0.001***	< 0.001***	0.002**	-	-

*AUC* area under the receiver operating characteristic curve, *ASAS* Assessment of SpondyloArthritis International Society

* *p* < 0.05; ** *p* < 0.01; *** *p* < 0.001

### Model interpretation

**Fig. 6:**
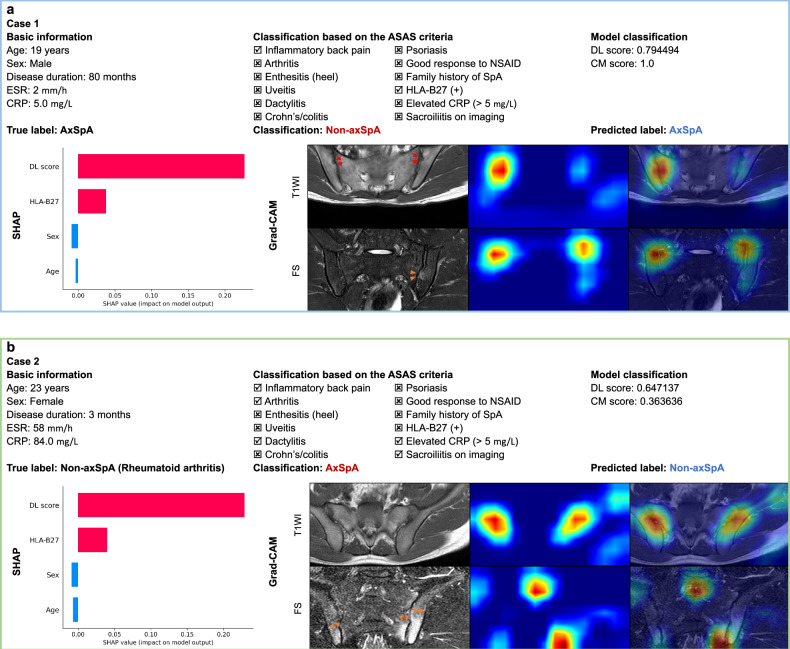
Representative cases correctly classified by the combined model. **a** A 19-year-old male with axSpA was misclassified as non-axSpA based on the ASAS classification criteria, but the combined model correctly reclassified him as axSpA. **b** A 23-year-old female with non-axSpA (rheumatoid arthritis) was misclassified as axSpA, but the combined model correctly reclassified her as non-axSpA. SHAP values are presented to indicate the contributions of various variables, with red highlighting potential risk factors for axSpA and blue denoting protective features. MRI heatmaps were generated using Grad-CAM, with red areas representing highly activated regions. The red arrowheads indicate bone erosion. The orange arrowheads indicate bone marrow edema. Text in blue denotes correct classifications, while text in red denotes incorrect classifications. ASAS, the Assessment of SpondyloArthritis International Society; axSpA, axial spondyloarthritis; CRP, C-reactive protein; ESR, erythrocyte sedimentation rate; HLA, human leukocyte antigen; DL, deep learning; CM, combined model; T1WI, T1-weighted imaging; FS, fluid-sensitive fat suppression; Grad-CAM, gradient-weighted class activation mapping; SHAP, Shapley additive explanation

To elucidate the decision-making process of the combined model, we employed SHAP [18] and Grad-CAM [17] for visualization (Figs. 6 and S4). Grad-CAM demonstrated that the DL model effectively focused on lesions within the bilateral SIJs. Meanwhile, SHAP analysis confirmed that the DL score had the greatest impact on axSpA identification, while the importance of clinical variables varied across patients.

## Discussion

In this study, we developed an ML combined model for differentiating axSpA and non-axSpA by integrating the DL score based on SIJ MRI and clinical risk factors. The model demonstrated strong performance across various testing phases, including internal and external testing as well as subgroup analyses. Notably, our model outperformed the ASAS classification criteria in diagnostic performance (AUC, 0.858 vs. 0.650, *p* < 0.001), offering potential improvements in axSpA diagnosis and more effective treatment strategies.

Data-driven approaches, particularly those utilizing DL models, are increasingly crucial for the accurate identification of SIJ lesions. Traditional 2D ResNet-based architectures have been effective in classifying inflammatory and structural damage indicative of axSpA [13, 19, 20]. Our DL model, however, advanced these efforts by employing a 3D ResNet, developed based on T1WI and FS sequences recommended by the ASAS MRI working group [21]. The 3D model’s advantage lies in capturing spatial relationships across MRI slices [22], crucial for identifying axSpA lesions such as bone marrow edema and sclerosis that often span multiple SIJ slices [23]. Our model achieved a strong AUC of 0.837 for reliably differentiating SIJ lesions suggestive of axSpA from non-axSpA on the internal test set. Although its performance slightly declined on external datasets, it remained robust during prospective validation. This discrepancy might arise from variations in imaging protocols since the prospective dataset and the internal test set originated from the same center. Our combined model, built on the KNN-11 algorithm’s flexibility, improved generalization by considering multiple neighbors, thus enhancing noise tolerance [24]. It successfully combined clinical risk factors (age, sex, and HLA-B27 status) with MRI findings, achieving AUCs from 0.780 to 0.912 across test sets, highlighting the integration between imaging and clinical data in axSpA diagnosis.

Delays in diagnosing axSpA using the traditional ASAS criteria are common [9], but our model improved this with higher sensitivity (87.8% vs. 42.4%, *p* < 0.001) and accuracy (78.7% vs. 56.9%, *p* < 0.001). It effectively identified axSpA often missed due to rigid ASAS adherence. For example, cases exhibiting only two SpA features, such as the presence of IBP and positive HLA-B27, may be misclassified if additional SpA features or sacroiliitis on imaging are absent according to the ASAS criteria [7]. However, subtle SIJ lesions might be overlooked, leading to misclassification as non-axSpA. Our model provided correct classifications in such cases (Fig. 6a), potentially reducing misdiagnoses caused by clinician fatigue and strict ASAS adherence. Additionally, the clinician might occasionally misinterpret SIJ lesions in non-axSpA patients as those indicative of axSpA due to overlapping features in both conditions [25]. Our model minimized these misinterpretations by incorporating additional information beyond MRI and HLA-B27 status, including age and sex (Fig. 6b). These two variables also assisted in diagnosing axSpA, as the disease is more commonly observed in young male patients [26, 27]. When our model’s results differ from the ASAS classification, clinicians are advised to exercise increased vigilance and consider supplementary diagnostic tests to reduce the risk of misdiagnosis.

To enhance the clinical application of our combined model, it is imperative to clarify the model’s decision process, especially in error cases, to foster transparency and trust [28]. Our SHAP [18] analysis indicated that the MRI-based DL score significantly impacts predictive outcomes (Fig. S4). Additionally, HLA-B27 was highlighted as a key predictor for axSpA, aligning with its importance in the ASAS criteria [7]. Furthermore, age and sex also showed an impact on model predictions, reflecting previous research on the demographic association of axSpA [26, 27]. For misdiagnosed cases, Grad-CAM [17] analysis revealed that the DL score sometimes relied on irrelevant regions like subcutaneous fat, the spinal canal, or vascular structures (Fig. S4), potentially due to similarities in MRI signals with axSpA lesions [21]. By combining SHAP with Grad-CAM visualizations, clinicians can more easily spot which clinical variables or image regions the model emphasizes, reinforcing the importance of a careful and comprehensive diagnostic approach.

Analyzing diagnostic performance across subgroups, our model showed promise in older populations (AUC of 0.838 for patients over 31), addressing a gap where the ASAS criteria do not apply to individuals over 45 [7]. Additionally, it excelled in identifying early-stage axSpA cases, traditionally prone to diagnostic delays [29], with a noteworthy AUC of 0.849, underscoring the potential to substantially reduce diagnostic delay. Importantly, while most previous studies have focused on European populations [20], our model was validated in Asian datasets, where HLA-B27-negative patients are more prevalent [30]. This is crucial, as HLA-B27-negative axSpA patients often experience diagnostic delays due to a lack of awareness of their symptoms [31]. Our model effectively addressed the diagnostic challenge for analyzing HLA-B27-negative patients (AUC, 0.722). However, it showed limitations in HLA-B27-positive patients (AUC, 0.570) due to sample imbalance, with only 6 HLA-B27-positive non-axSpA cases. More extensive validation is needed to fully assess the model’s capabilities in this subgroup.

This study had several limitations. First, despite using a multicenter dataset, our model’s training and testing relied mainly on data from a single center (center A). The external dataset, limited in size and predominantly Asian, may reduce the model’s generalizability to broader populations, underscoring the need for validation in more diverse cohorts. Second, our multivariate analysis included only six clinical variables (age, sex, disease duration, CRP, ESR, HLA-B27). However, diagnosing axSpA often requires considering a wider range of clinical factors, such as medical history and lifestyle. Future studies should incorporate more comprehensive clinical variables to enhance diagnostic accuracy. Third, the use of manual bounding boxes to identify SIJ lesions, though straightforward, is labor-intensive. Future research should integrate automated SIJ region recognition into the model to improve annotation efficiency and clinical utility.

## Conclusions

This study demonstrated that an ML approach leveraging both SIJ MRI and clinical risk factors can outperform traditional classification criteria like the ASAS in identifying axSpA. The ML combined model’s high sensitivity and accuracy, coupled with its interpretability, make it a promising tool for early and accurate diagnosis, which is critical for optimizing treatment and improving long-term outcomes in axSpA patients.

## Supplementary information

ELECTRONIC SUPPLEMENTARY MATERIAL

## Data Availability

Data generated or analyzed during the study are available from the corresponding author by request.

## Abbreviations

ASAS: Assessment of SpondyloArthritis International Society
AUC: Area under the receiver operating characteristic curve
AxSpA: Axial spondyloarthritis
CRP: C-reactive protein
DL: Deep learning
ESR: Erythrocyte sedimentation rate
FS: Fluid-sensitive fat suppression
Grad-CAM: Gradient-weighted class activation mapping
HLA: Human leukocyte antigen
IBP: Inflammatory back pain
KNN: K-nearest-neighbors
ML: Machine learning
NSAIDs: Nonsteroidal anti-inflammatory drugs
ROC: Receiver operating characteristic curve
SHAP: Shapley additive explanation
SIJs: Sacroiliac joints
SpA: Spondyloarthritis
T1WI: T1-weighted imaging
T2WI: T2-weighted imaging
