# Supplementary material for: Enhanced diagnosis of axial spondyloarthritis using machine learning with sacroiliac joint MRI: a multicenter study
Source: Insights Imaging. 2025 Apr 25;16:91. doi: 10.1186/s13244-025-01967-x (PMC12031678; doi:10.1186/s13244-025-01967-x)
Supplement: Supplementary file 1 — ELECTRONIC SUPPLEMENTARY MATERIAL [file 13244_2025_1967_MOESM1_ESM.pdf]

Enhanced diagnosis of axial spondyloarthritis using machine leaning with sacroiliac joint MRI: a multicenter study

ELECTRONIC SUPPLEMENTARY MATERIAL

Supplementary Appendix S1: Inclusion and exclusion criteria

Eligible patients met the following inclusion criteria: (A) a diagnosis of axial spondyloarthritis (axSpA) or non-axSpA confirmed by rheumatologists at the initial visit or after at least one year of follow-up, accompanied by chronic low back pain; (B) MRI and laboratory analyses conducted within two weeks prior to the initiation of treatment; and (C) availability of complete clinical information in electronic medical records.

Exclusion criteria included: (A) concurrent diagnoses of both axSpA and non-axSpA; (B) prior treatment with biologic disease-modifying anti-rheumatic drugs (including tumor necrosis factor inhibitors, CD20 monoclonal antibodies, and interleukin antagonists) before undergoing MRI; and (C) poor MRI quality, as evaluated by a senior musculoskeletal radiologist (Q.Y., with 15 years of experience), due to artifacts, missing sequences, or incomplete sacroiliac joint slices.

Supplementary Appendix S2: Diagnostic assessment

The axSpA was determined through a two-step process. First, two musculoskeletal radiologists independently evaluated sacroiliac joint changes on radiographs or MRI, following the modified New York criteria and ASAS MRI guidelines [1; 2]. The radiologists had access to patients' age, sex, and disease duration but were blinded to other clinical features. Any discrepancies in their assessments were resolved through discussion. Second, two rheumatologists reviewed imaging and clinical features in reference to the ASAS classification criteria [3]. They also considered additional factors such as infection indicators, rheumatoid factor, uric acid, pregnancy status, and physical measurements (e.g., spinal flexion, Mennell's test, Patrick's test) to establish the final diagnosis. Any differences in their assessments were resolved by consensus, with the rheumatologists' final diagnosis (axSpA and non-axSpA) considered the reference standard.

Supplementary Appendix S3: Details of non-axial spondyloarthritis

Non-axSpA patients in the internal dataset (center A) were as follows: adult-onset Still's disease (1 case), Behcet's disease (1 case), bone metastases (1 case), chronic myelofibrosis (1 case), condensing osteitis (11 case), condensing osteitis (1 case), connective tissue diseases (5 cases), degenerative arthritis (29 cases), gouty arthritis (17 cases), hip arthritis (2 cases), IgA nephropathy (1 case), infectious sacroiliitis (4 cases), juvenile idiopathic arthritis (15 cases), juvenile rheumatoid arthritis (1 case), kidney stone (1 case), nonspecific low back pain (94 cases), plasma cell disorders (3 cases), polyarthritis (1 case), psoriatic arthritis (5 cases), reactive arthritis (6 cases), rheumatoid arthritis (22 cases), sacroiliac joint suppurative osteomyelitis (2 cases), sacroiliac joint tuberculosis (1 case), systemic lupus erythematosus (5 cases), type 1 diabetes (1 case), undifferentiated arthritis (32 cases), undifferentiated arthritis； condensing osteitis (1 case), undifferentiated connective tissue disease (12 cases), and undifferentiated spondyloarthritis (3 cases).

Non-axSpA patients in the external dataset I (center B) were as follows: gouty arthritis (2 cases) and nonspecific low back pain (17 cases).

Non-axSpA patients in the external dataset II (center C) were as follows: gouty arthritis (1 case), psoriatic arthritis (1 case), condensing osteitis (2 cases), undifferentiated spondyloarthritis (1 case), and nonspecific low back pain (14 cases).

Non-axSpA patients in the external dataset III (center D) were as follows: condensing osteitis (2 cases), degenerative arthritis (6 cases), fibromyalgia (2 cases), gouty arthritis (1 case), hypophosphatemic osteomalacia (1 case), lumbar abscess (1 case), Mycobacterium kansasii infection (1 case), nonspecific low back pain (7 cases), psoriatic arthritis (2 cases), rheumatoid arthritis (1 case), Sjögren's syndrome (1 case), systemic lupus erythematosus (1 case), and undifferentiated arthritis (1 case).

Non-axSpA patients in the prospective validation dataset (center A) were as follows: avascular necrosis of the femoral head (1 case), Brucellosis spondylitis (1 case), chronic renal insufficiency (1 case), condensing osteitis (2 cases), connective tissue disease (2 cases), degenerative arthritis (7 cases), gouty arthritis (1 case), juvenile idiopathic arthritis (4 cases), monoarthritis (1 case), nonspecific low back pain (16 cases), osteoporosis (1 case), Perthes disease (1 case), polyarthritis (6 cases), polymyalgia rheumatica (1 case), psoriatic arthritis (1 case), rheumatoid arthritis (2 cases), systemic lupus erythematosus (1 case), undifferentiated arthritis (4 cases), and undifferentiated connective tissue disease (1 case).

Supplementary Appendix S4: Image preprocessing

All MRI images underwent preprocessing for consistency and reliability. First, N4 bias field correction was applied to mitigate intensity inhomogeneities [4]. Subsequently, the images were resampled to achieve an in-plane pixel size of 1.0 mm × 1.0 mm. After resampling, images were randomly cropped to dimensions of 12 × 256 × 512 around the bounding box and uniformly normalized to a range of [−1, 1].

Supplementary Appendix S5: Model architecture

In this study, we did not utilize transfer learning and instead trained our model from scratch using a modified 3D ResNet50 architecture. This decision was based on the following considerations: 1) Feature Distribution Differences: The feature distributions in volumetric medical images differ significantly from natural images. While natural image datasets typically cover a broad spectrum of categories, medical imaging datasets for specific diagnostic tasks have a much narrower distribution. For instance, in our task of disease diagnosis, the macroscopic anatomical structures across subjects are highly similar, and the challenge lies in identifying subtle differences within these structures. This fundamental difference diminishes the utility of transfer learning from natural image datasets. 2) Pretrained Weights Compatibility: Existing pretrained ResNet models are primarily 2D and derived from natural image datasets (e.g., ImageNet). Adapting these 2D pretrained weights to our customized 3D ResNet architecture requires complex weight conversion techniques, such as weight slicing, inflation, or merging strategies [5; 6]. Each of these methods introduces additional design choices and evaluation overhead, which extends beyond the scope of this study. 3) Architecture Specialization: Our modified 3D ResNet architecture is tailored for 2D MRI images with anisotropic voxel spacing. Specifically, our model applies lower downsampling frequency along the through-plane direction (slice thickness), which often has voxel spacing several times larger than the in-plane resolution. This design ensures that the receptive field of deep feature maps remains closer to isotropic, promoting consistency at the physical level. Such architectural modifications diverge from mainstream medical image pretraining paradigms, such as MedicalNet [7], which simply replaces all 2D convolutional kernels with their 3D counterparts to construct general 3D ResNet structures. These differences further complicate the direct application of transfer learning.

Supplementary Appendix S6: Model training and selection

Each deep learning (DL) underwent fivefold cross-validation on the training set for model validation and epoch selection. To maintain balanced proportions of positive and negative samples across the patient dataset, each fold was stratified accordingly. To address class imbalance, the binary cross-entropy loss function was employed with class weights adjusted inversely proportional to the frequency of each class. Optimal model parameters were selected based on achieving the highest area under the receiver operating characteristic curve during validation. For testing purposed, outputs from the fivefold cross-validation models were averaged. The DL models were trained for 200 epochs per fold using the AdamW [8] optimizer, with a learning rate of 0.0003, moving average coefficients β_1 of 0.9, β_2 of 0.999, a weight decay coefficient of 0.01. Additionally, a learning rate scheduler is applied where the learning rate remains constant for the first 100 epochs and then decays progressively by multiplying it by a factor of 0.99 for each subsequent epoch. The mini-batch-size is set to 8. The binary cross-entropy loss function was utilized.

We observed that while the validation AUC steadily increases with training epochs, the validation loss exhibited a gradual upward trend (Supplementary Fig. S5). This discrepancy primarily aroused from the inherent difference between AUC and cross-entropy loss: AUC measures the model's ability to correctly rank positive and negative samples, focusing on classification ranking performance, whereas cross-entropy loss emphasizes the accuracy and confidence of predicted probabilities. Consequently, improvements in ranking can occur even as predicted probabilities become more confident or extreme, resulting in higher loss values. Despite the observed increase in validation loss, the ongoing improvement in AUC, along with other metrics such as F1 score and accuracy, demonstrates that the model maintained robust discriminatory power throughout training. To mitigate potential overfitting, we employed standard regularization techniques, including weight decay and early stopping, to ensure training stability and generalization. During cross-validation, we selected models or applied early stopping based on the iteration where the validation AUC reached its peak for each fold. This strategy ensured that the early stopping mechanism halts training at a relatively optimal point. Notably, this approach

aligned with the model selection strategy proposed by nnUNet [9], which emphasizes choosing the model checkpoint with the best validation performance to enhance generalizability. These evidences suggest that while the model shows a slight overfitting tendency on the validation set, its classification performance remains effective and reliable.

Regarding the sufficiency of a 200-epoch training period, Supplementary Fig. S5 demonstrates that this duration is adequate. Notably, when employing our model selection strategy (early stopping based on peak AUC in the validation set), the majority of folds achieved their optimal AUC between 100–150 epochs, indicating stable convergence within the designated training epochs.

Supplementary Appendix S7: Model hyperparameter optimization

To address concerns regarding hyperparameter optimization, a systematic grid search experiment was conducted to evaluate the impact of different learning rates on model performance. The search range spanned from 1×10^{-5} to 3×10^{-2}, with results illustrated in Supplementary Fig. S6. The results demonstrate that a learning rate of 3×10^{-4}, which was initially adopted in the main experiments based on conventional practices, achieves stable and optimal performance in terms of AUC, loss, and accuracy metrics. Specifically, learning rates lower than 1×10^{-5} resulted in slower convergence and suboptimal performance, while higher learning rates such as 3×10^{-2} led to training instability and performance degradation. These findings validate the appropriateness of the originally selected learning rate of 3×10^{-4}, highlighting its robustness for this task.

Supplementary Appendix S8: Experimental environment

The experimental environment utilized for data preprocessing and model implementation was based on Python (version 3.10.10) running on an Ubuntu 18.04 LTS operating system. The hardware configuration included an Intel Xeon E5-2650 v4 CPU and a Nvidia GeForce RTX 3090 GPU. Specifically, the following open-source Python packages were employed: SimpleITK (version 2.3.1) for N4 bias field correction in medical imaging data; MiceForest (version 3.1.1) for performing multiple imputation of missing clinical information; Scikits-Bootstrap (version 1.1.0) for bootstrap resampling procedures; Scikit-Learn (version 1.3.2)

for calculating metrics related to machine learning models; and Pytorch (version 2.1.2) served as our deep learning framework.

Supplementary Appendix S9: Details of model interpretation

Gradient-weighted class activation mapping (Grad-CAM) [10] was utilized to evaluate gradient information from the final convolutional layer of the deep learning model. This method allows us to map the gradients back to the original input dimensions, thereby identifying image regions that significantly influence the model's decision-making process. For the machine learning combined model, we applied Shapley additive explanation (SHAP) [11] values to elucidate the contribution of each variable. This approach helps in explaining the prediction results and providing a measure of feature importance.

Supplementary Appendix S10: Exploration of improved performance of the MRI-based deep learning model

The initial specificity of the MRI-based DL model using a default cutoff of 0.5 on external test sets I, II, and III ranged from 31.6% to 47.4% (Table 1). Optimization of the cutoff based on the Youden index prioritized specificity (57.9%–77.8%), albeit at the cost of low sensitivity (42.3%–54.4%). Aiming to enhance specificity, we selected cutoff values that maintained a specificity above 0.7 on the internal test set. The optimal cutoff value of 0.592 achieved a specificity of 0.75, balancing sensitivity (65.4%–74.0%) and specificity (51.9%–52.6%) across external test sets.

Supplementary Tables

Supplementary Table S1 Definition of SpA features [3]

SpA feature	Definition
IBP	IBP according to experts[12]:14 at least four out of five parameters present: (1) age at onset < 40 years; (2) insidious onset; (3) improvement with exercise; (4) no improvement with rest; (5) pain at night (with improvement upon getting up)
Arthritis	Past or present active synovitis diagnosed by a physician
Enthesitis (heel)	Heel enthesitis: past or present spontaneous pain or tenderness at examination of the site of the insertion of the Achilles tendon or plantar fascia at the calcaneus
Uveitis	Past or present uveitis anterior, confirmed by an ophthalmologist
Dactylitis	Past or present dactylitis, diagnosed by a physician
Psoriasis	Past or present psoriasis, diagnosed by a physician
IBD	Past or present Crohn’s disease or ulcerative colitis diagnosed by a physician
Good response to NSAID	24–48 h after a full dose of a NSAID the back pain is not present any more or is much better
Family history of SpA	Presence in first-degree (mother, father, sisters, brothers, children) or second-degree (maternal and paternal grandparents, aunts, uncles, nieces and nephews) relatives of any of the following: (1) AS; (2) psoriasis; (3) acute uveitis; (4) reactive arthritis; (5) IBD
Elevated CRP	CRP concentration above upper normal limit in the presence of back pain, after exclusion of other causes for elevated CRP concentration
HLA-B27	Positive testing according to standard laboratory techniques
Sacroiliitis by MRI	Active inflammatory lesions of sacroiliac joints with definite bone marrow oedema/osteitis, suggestive of sacroiliitis associated with SpA

SpA **spondyloarthritis,** ***AS*** **ankylosing spondylitis,** ***IBP*** **inflammatory back pain,** ***IBD*** **inflammatory bowel disease,** ***NSAID*** **non-steroidal anti-inflammatory drug,** ***CRP*** **C-reactive protein,** ***HLA*** **human leukocyte antigen**

Supplementary Table S2 Detailed of MRI protocols

Center	Scanner	Sequence	TR/TE (ms)	Slice thickness (mm)	Slice gap (mm)	FOV (mm)	Matrix size
Center A	Philips 1.5T (Achieva)	Coronal T1W	500/18	4.0	0.4	143	396 × 318
		Axial T2WI	3000/100	4.0	0.4	140	418 × 320
		Coronal T2WI	5228/100	4.0	0.4	139	400 × 310
		Axial PDWI SPAIR	2586/30	6.0	0.6	140	336 × 264
		Coronal T2WI SPAIR	3000/100	4.0	0.4	126	418 × 320
		Coronal PDWI SPAIR	2898/30	4.0	0.4	132	348 × 270
		Coronal STIR	2400/60	3.0	0.3	150	300 × 238
	Philips 3.0T (Ingenia)	Axial T1WI	664/20	5.0	0.5	175	476 × 351
		Coronal T1WI	550/22	3.0	0.3	140	380 × 336
		Axial T2WI	4565/90	5.0	0.5	133	400 × 308
		Coronal T2WI	2300/85	3.0	0.3	139	400 × 392
		Axial T2WI SPAIR	4230/70	6.0	0.6	175	412 × 405
		Coronal T2WI SPAIR	2929/70	3.0	0.3	139	400 × 382
Center B	Siemens 1.5T (Avanto)	Axial T1WI	615/11	5.0	0.5	100	384 × 307
		Coronal T1WI	450/22	3.0	0.3	100	256 × 243
		Axial T2WI	3000/94	4.0	0.4	75	320 × 216
		Coronal T2W Dixon water-only	4800/44	3.0	0.3	100	256 × 230
	GE 1.5T	Coronal T1WI	460/18	4.0	0.4	100	512 × 192

	(Genesis Signa)	Axial T2WI SPECIAL	4800/63	5.0	0.5	75	448 × 192
	Philips 3.0T (Ingenia)	Axial T1WI	550/20	4.0	0.4	100	423 × 353
		Coronal STIR	3680/90	3.0	0.3	100	390 × 410
Center C	Philips 3.0T (Achieva)	Axial T1WI	500/20	5.0	0.5	150	427 × 361
		Coronal T2WI SPAIR	1970/70	4.0	0.4	140	320 × 253
		Coronal STIR	3010/60	5.0	0.5	147	348 × 260
	Philips 3.0T (Ingenia)	Axial T1WI	540/20	4.0	0.4	116	471 × 427
		Coronal T2WI SPAIR	3500/120	4.0	0.4	125	468 × 275
Center D	GE 1.5T (Optima MR360)	Coronal T1WI	570/15	3.0	0.3	100	384 × 224
		Coronal T2WI SPECIAL	3070/90	3.0	0.3	100	320 × 192
	Philips 1.5T (Achieva)	Coronal T1WI	500/20	3.0	0.3	100	320 × 252
		Coronal T2WI SPAIR	4430/70	3.0	0.3	116	247 × 198
	GE 3.0T (Signa Architect)	Coronal T1WI	420/10	3.0	0.3	100	320 × 256
		Coronal T2WI SPECIAL	3310/84	3.0	0.3	100	320 × 224
	GE 3.0T (Discovery MR750)	Coronal T1WI	520/15	3.0	0.3	100	320 × 224
		Coronal T2WI SPECIAL	4000/65	3.0	0.3	100	320 × 256
	Siemens 3.0T (Prisma)	Coronal T1WI	670/11	3.0	0.3	100	256 × 256
		Coronal T2W	3410/82	3.0	0.3	100	256 × 256

	Dixon water-only					
	Coronal STIR	3620/57	3.0	0.3	100	256 × 256
United Imaging 3.0T (uMR 790)	Coronal T1WI	360/10	3.0	0.3	100	320 × 224
	Coronal T2W WFI water-only	2872/87	3.0	0.3	100	320 × 256

ms **millisecond,** ***mm*** **millimeter,** ***TR*** **time of repetition,** ***TE*** **time of echo,** ***FOV*** **field of view,** ***T1WI*** **T1-weighted imaging,** ***T2WI*** **T2-weighted imaging,** ***SPAIR*** **spectral attenuated inversion recovery,** ***PDW*** **proton density weighted,** ***STIR*** **short tau inversion recovery,** ***SPECIAL*** **spectral inversion at lipids,** ***WFI*** **water-fat imaging**

Supplementary Table S3 Performance of single- and multiple-sequence MRI-based deep learning model on the internal test set

Model	AUC	Accuracy	Sensitivity	Specificity	F1 Score	Precision
T1WI	0.799<br>(0.720–0.867)	76.4%<br>(70.2%–82.6%)	80.3%<br>(72.8%–87.4%)	67.9%<br>(55.3%–79.7%)	82.4%<br>(76.6%–87.7%)	84.5%<br>(77.0%–90.6%)
T2WI	0.798<br>(0.724–0.864)	72.5%<br>(65.7%–79.2%)	72.1%<br>(62.9%–79.5%)	73.2%<br>(60.8%–84.3%)	78.2%<br>(71.9%–83.9%)	85.4%<br>(78.4%–92.2%)
FS	0.778<br>(0.697–0.844)	73.0%<br>(66.8%–79.8%)	79.5%<br>(72.6%–86.0%)	58.9%<br>(45.8%–71.7%)	80.2%<br>(74.2%–85.5%)	80.8%<br>(73.6%–87.2%)
T1WI+T2WI	0.828<br>(0.766–0.888)	74.7%<br>(68.0%–80.9%)	78.7%<br>(71.1%–85.4%)	66.1%<br>(52.5%–77.8%)	81.0%<br>(75.7%–86.4%)	83.5%<br>(76.4%–90.0%)
T1WI+FS	0.837<br>(0.778–0.891)	77.5%<br>(71.3%–83.7%)	84.4%<br>(78.0%–90.4%)	62.5%<br>(50.0%–75.5%)	83.7%<br>(78.4%–88.2%)	83.1%<br>(76.0%–89.4%)
T2WI+FS	0.824<br>(0.757–0.884)	74.7%<br>(68.0%–80.9%)	77.0%<br>(69.7%–84.2%)	69.6%<br>(58.1%–81.0%)	80.7%<br>(74.7%–86.3%)	84.7%<br>(77.7%–91.1%)
T1WI+T2WI+FS	0.846<br>(0.789–0.902)	75.8%<br>(69.7%–82.0%)	81.1%<br>(74.2%–88.1%)	64.3%<br>(50.9%–76.7%)	82.2%<br>(76.8%–87.1%)	83.2%<br>(76.6%–90.1%)

The results were calculated with a cutoff of 0.5

AUC **area under the receiver operating characteristic curve,** ***T1WI*** **T1-weighted imaging,** ***T2WI*** **T2-weighted imaging,** ***FS*** **fluid-sensitive fat suppression**

Supplementary Table S4 Performance of the MRI-based deep learning model with different cutoff values

Model	AUC	Accuracy	Sensitivity	Specificity	F1 Score	Precision
Default (cutoff value of 0.5)						
Internal test set	0.837	77.5%	84.4%	62.5%	83.7%	83.1%
	(0.771–0.891)	(70.8%–83.7%)	(78.0%–90.4%)	(49.1%–74.5%)	(78.6%–88.4%)	(76.0%–89.6%)
External test set I	0.636	66.7%	92.3%	31.6%	76.2%	64.9%
	(0.452–0.800)	(53.3%–80.0%)	(80.6%–100.0%)	(10.5%–55.6%)	(62.1%–86.6%)	(48.6%–80.6%)
External test set	0.724	76.8%	88.0%	47.4%	84.6%	81.5%
II	(0.589–0.847)	(66.7%–87.0%)	(78.4%–96.1%)	(26.3%–68.8%)	(76.0%–91.4%)	(70.4%–90.7%)
External test set	0.710	72.6%	89.5%	37.0%	81.6%	75.0%
III	(0.594–0.822)	(61.9%–82.1%)	(81.1%–96.4%)	(19.0%–55.6%)	(74.1%–88.5%)	(64.5%–84.9%)
Cutoff value with best Youden index (cutoff value of 0.698)						
Internal test set	0.837	70.8%	60.7%	92.9%	74.0%	94.9%
	(0.773–0.894)	(64.6%–77.5%)	(51.8%–68.9%)	(85.7%–98.3%)	(67.0%–80.4%)	(89.7%–98.8%)
External test set I	0.636	48.9%	42.3%	57.9%	48.9%	57.9%
	(0.468–0.789)	(35.6%–64.4%)	(23.8%–61.5%)	(37.5%–80.0%)	(27.0%–66.7%)	(35.3%–78.9%)
External test set	0.724	53.6%	48.0%	68.4%	60.0%	80.0%
II	(0.588–0.858)	(40.6%–66.7%)	(34.6%–62.5%)	(47.6%–88.2%)	(46.7%–71.3%)	(65.5%–92.9%)
External test set	0.710	61.9%	54.4%	77.8%	66.0%	83.8%
III	(0.598–0.816)	(51.2%–72.6%)	(42.6%–67.3%)	(62.5%–92.6%)	(53.5%–75.8%)	(70.6%–94.6%)
Cutoff value with specificity of 70% (cutoff value of 0.578)						
Internal test set	0.837	75.3%	77.0%	71.4%	81.0%	85.5%
	(0.770–0.894)	(68.5%–80.9%)	(69.1%–84.0%)	(58.8%–83.0%)	(74.8%–86.1%)	(78.3%–91.8%)
External test set I	0.636	57.8%	65.4%	47.4%	64.2%	63.0%
	(0.454–0.791)	(44.4%–71.1%)	(44.8%–83.3%)	(26.3%–70.6%)	(46.1%–77.8%)	(45.8%–81.8%)
External test set	0.724	69.6%	76.0%	52.6%	78.4%	80.9%

II	(0.585–0.856)	(59.4%–79.7%)	(63.6%–86.8%)	(29.4%–76.5%)	(68.1%–86.5%)	(68.7%–91.5%)
External test set	0.710	66.7%	73.7%	51.9%	75.0%	76.4%
III	(0.596–0.828)	(56.0%–76.2%)	(61.8%–85.0%)	(31.8%–70.8%)	(64.7%–83.3%)	(65.5%–88.0%)
Cutoff value with specificity of 75% (cutoff value of 0.592)						
Internal test set	0.837	76.4%	77.0%	75.0%	81.7%	87.0%
	(0.776–0.896)	(70.2%–82.0%)	(69.7%–84.0%)	(63.3%–86.4%)	(75.4%–86.7%)	(80.4%–93.3%)
External test set I	0.636	60.0%	65.4%	52.6%	65.4%	65.4%
	(0.470–0.793)	(46.7%–75.6%)	(48.0%–84.0%)	(30.0%–73.7%)	(48.8%–78.7%)	(45.8%–84.6%)
External test set	0.724	68.1%	74.0%	52.6%	77.1%	80.4%
II	(0.578–0.850)	(56.5%–79.7%)	(61.4%–85.7%)	(28.6%–75.0%)	(66.7%–85.4%)	(68.4%–91.1%)
External test set	0.710	66.7%	73.7%	51.9%	75.0%	76.4%
III	(0.589–0.821)	(56.0%–76.2%)	(62.3%–84.7%)	(32.3%–70.4%)	(64.8%–83.2%)	(65.5%–87.0%)
Cutoff value with specificity of 80% (cutoff value of 0.635)						
Internal test set	0.837	74.2%	71.3%	80.4%	79.1%	88.8%
	(0.775–0.893)	(67.4%–80.3%)	(62.7%–79.3%)	(68.7%–90.0%)	(73.1%–84.7%)	(82.3%–94.5%)
External test set I	0.636	55.6%	57.7%	52.6%	60.0%	62.5%
	(0.455–0.796)	(40.0%–68.9%)	(37.5%–74.1%)	(29.4%–73.7%)	(42.5%–75.4%)	(42.9%–82.1%)
External test set	0.724	63.8%	64.0%	63.2%	71.9%	82.1%
II	(0.573–0.845)	(52.2%–75.4%)	(50.0%–77.4%)	(40.0%–84.2%)	(61.0%–82.1%)	(69.2%–93.0%)
External test set	0.710	64.3%	63.2%	66.7%	70.6%	80.0%
III	(0.593–0.820)	(53.6%–75.0%)	(50.0%–75.4%)	(47.6%–83.9%)	(59.8%–80.0%)	(66.7%–90.7%)

***AUC* area under the receiver operating characteristic curve**

Supplementary Table S5 Performance of MRI-based deep learning model in subgroup analysis

Subgroup	AUC	Accuracy	Sensitivity	Specificity	F1 Score	Precision
Age						
< 31 years	0.756	81.9%	93.0%	27.6%	89.5%	86.3%
	(0.662–0.837)	(76.0%–87.7%)	(88.4%–96.6%)	(12.9%–44.8%)	(85.9%–93.0%)	(80.6%–91.6%)
≥ 31 years	0.726	69.3%	79.6%	56.5%	74.1%	69.2%
	(0.655–0.792)	(62.4%–75.1%)	(71.9%–87.2%)	(46.4%–66.3%)	(68.0%–79.8%)	(60.7%–76.9%)
Sex						
Male	0.782	78.8%	89.6%	42.4%	86.7%	84.1%
	(0.719–0.842)	(73.8%–83.8%)	(85.3%–93.6%)	(30.6%–55.9%)	(82.9%–90.0%)	(79.2%–88.5%)
Female	0.681	66.4%	77.8%	56.5%	68.3%	60.9%
	(0.586–0.780)	(57.8%–75.0%)	(67.2%–88.5%)	(44.6%–69.6%)	(58.3%–77.0%)	(50.0%–72.2%)
Disease duration						
< 2 years	0.774	73.1%	89.0%	53.9%	78.3%	69.8%
	(0.698–0.845)	(67.1%–79.6%)	(81.9%–94.9%)	(42.9%–65.7%)	(71.3%–84.1%)	(61.5%–78.6%)
≥ 2 years	0.752	76.6%	86.0%	42.2%	85.2%	84.4%
	(0.672–0.824)	(70.8%–81.8%)	(80.7%–90.9%)	(27.7%–56.3%)	(81.0%–89.0%)	(79.1%–89.7%)
HLA-B27 status						
(+)	0.699	87.1%	89.4%	16.7%	93.1%	97.0%
	(0.523–0.859)	(82.3%–91.9%)	(84.6%–93.9%)	(0.0%–50.0%)	(90.5%–95.8%)	(94.0%–99.4%)
(-)	0.660	59.5%	79.2%	45.6%	61.8%	50.7%
	(0.559–0.765)	(50.8%–68.1%)	(67.3%–90.4%)	(33.8%–57.6%)	(51.7%–71.2%)	(38.9%–62.3%)
Unknown	0.794	68.9%	85.2%	59.6%	66.7%	54.8%
	(0.693–0.891)	(58.1%–78.4%)	(69.7%–96.7%)	(44.4%–72.7%)	(51.6%–78.7%)	(40.9%–69.0%)

***AUC* area under the receiver operating characteristic curve**

Supplementary Table S6 Univariate analysis and multivariate analysis for clinical risk factors

**Clinical Feature**	**Univariate**	***p***	**Multivariate**	***p***
Age	0.945 (0.933, 0.957)	<0.001	0.968 (0.952, 0.984)	<0.001
Sex		<0.001		0.044
Female	Reference		Reference	
Male	2.646 (1.904, 3.677)		1.683 (1.014, 2.795)	
Disease duration	1.002 (0.999, 1.004)	0.238		
ESR	0.999 (0.993, 1.006)	0.852		
CRP	1.006 (0.998, 1.014)	0.135		
HLA-B27	3.054 (2.482, 3.755)	<0.001	2.604 (1.931, 3.511)	<0.001

ESR **erythrocyte sedimentation rate,** ***CRP*** **C-reactive protein,** ***HLA*** **human leukocyte antigen**

Supplementary Table S7 Performance of combined models based on different algorithms

Model	AUC	Accuracy	Sensitivity	Specificity	F1 Score	Precision
Logistic regression						
Internal test set	0.852 (0.793–0.906)	79.2% (73.6%–84.8%)	88.5% (82.7%–93.7%)	58.9% (45.7%–72.3%)	85.4% (80.5%–89.5%)	82.4% (75.8%–88.6%)
External test set I	0.686 (0.518–0.836)	66.7% (51.1%–80.0%)	96.2% (87.5%–100.0%)	26.3% (8.3%–47.6%)	76.9% (64.3%–87.3%)	64.1% (48.6%–78.9%)
External test set II	0.768 (0.626–0.888)	81.2% (72.5%–89.9%)	96.0% (89.8%–100.0%)	42.1% (20.0%–65.0%)	88.1% (80.8%–94.1%)	81.4% (71.7%–90.6%)
External test set III	0.805 (0.709–0.890)	73.8% (64.3%–83.3%)	98.2% (94.3%–100.0%)	22.2% (7.7%–40.7%)	83.6% (75.6%–89.5%)	72.7% (62.8%–82.1%)
All external test sets	0.764 (0.697–0.831)	74.7% (68.2%–80.8%)	97.0% (93.7%–99.3%)	29.2% (18.9%–40.3%)	83.8% (78.9%–88.1%)	73.7% (67.1%–79.9%)
Naïve Bayes						
Internal test set	0.849 (0.791–0.900)	79.8% (73.6%–85.4%)	90.2% (85.1%–94.9%)	57.1% (43.9%–70.8%)	85.9% (81.1%–90.1%)	82.1% (75.4%–88.5%)
External test set I	0.679 (0.513–0.826)	64.4% (48.9%–75.6%)	96.2% (87.5%–100.0%)	21.1% (4.5%–40.0%)	75.8% (63.5%–86.5%)	62.5% (46.5%–76.3%)
External test set II	0.756 (0.618–0.878)	81.2% (72.5%–89.9%)	96.0% (88.9%–100.0%)	42.1% (21.1%–64.7%)	88.1% (80.4%–93.5%)	81.4% (71.9%–90.0%)
External test set III	0.768 (0.655–0.859)	73.8% (64.3%–82.1%)	98.2% (94.3%–100.0%)	22.2% (7.4%–38.1%)	83.6% (76.9%–89.7%)	72.7% (62.7%–81.7%)
All external test sets	0.742 (0.673–0.813)	74.2% (68.2%–79.8%)	97.0% (94.0%–99.3%)	27.7% (16.9%–38.9%)	83.5% (79.2%–87.7%)	73.3% (66.7%–79.8%)
KNN-7						
Internal test set	0.849	78.1%	88.5%	55.4%	84.7%	81.2%

	(0.785–0.905)	(71.9%–83.7%)	(82.9%–94.0%)	(42.1%–68.4%)	(79.7%–89.4%)	(74.2%–87.5%)
External test set I	0.895<br>(0.806–0.966)	77.8%<br>(64.4%–88.9%)	100.0%<br>(100.0%–100.0%)	47.4%<br>(25.0%–69.6%)	83.9%<br>(72.1%–92.8%)	72.2%<br>(56.8%–85.7%)
External test set II	0.796<br>(0.673–0.897)	75.4%<br>(65.2%–85.5%)	88.0%<br>(79.1%–96.1%)	42.1%<br>(20.0%–65.0%)	83.8%<br>(75.6%–90.6%)	80.0%<br>(68.5%–90.4%)
External test set III	0.928<br>(0.866–0.977)	83.3%<br>(76.2%–90.5%)	94.7%<br>(88.3%–100.0%)	59.3%<br>(37.9%–79.3%)	88.5%<br>(82.3%–94.2%)	83.1%<br>(73.0%–91.8%)
All external test sets	0.882<br>(0.829–0.923)	81.3%<br>(75.8%–86.9%)	88.0%<br>(82.3%–93.5%)	67.7%<br>(56.7%–79.6%)	86.3%<br>(81.9%–90.4%)	84.8%<br>(78.9%–90.7%)

AUC **area under the receiver operating characteristic curve,** ***KNN*** **K-nearest-neighbors**

Supplementary Table S8 Performance of KNN-based combined models using different K values with a cutoff of 0.5

Model	AUC	Accuracy	Sensitivity	Specificity	F1 Score	Precision
KNN-7						
Internal test set	0.849 (0.792–0.904)	78.1% (71.9%–84.3%)	88.5% (82.5%–93.6%)	55.4% (42.1%–67.9%)	84.7% (79.5%–89.0%)	81.2% (74.5%–87.5%)
External test set I	0.895 (0.792–0.965)	77.8% (64.4%–88.9%)	100.0% (100.0%–100.0%)	47.4% (25.0%–70.6%)	83.9% (73.3%–92.8%)	72.2% (56.8%–86.5%)
External test set II	0.796 (0.676–0.898)	75.4% (63.8%–85.5%)	88.0% (78.0%–96.2%)	42.1% (20.0%–66.7%)	83.8% (75.6%–91.4%)	80.0% (69.6%–90.3%)
External test set III	0.928 (0.862–0.979)	83.3% (75.0%–90.5%)	94.7% (88.7%–100.0%)	59.3% (40.0%–77.8%)	88.5% (82.5%–93.8%)	83.1% (73.1%–90.9%)
KNN-9						
Internal test set	0.851 (0.795–0.906)	81.5% (75.8%–87.1%)	87.7% (81.5%–93.1%)	67.9% (55.5%–80.4%)	86.6% (81.6%–90.6%)	85.6% (79.0%–91.3%)
External test set I	0.878 (0.773–0.961)	77.8% (64.4%–88.9%)	96.2% (87.5%–100.0%)	52.6% (28.6%–76.5%)	83.3% (72.4%–92.1%)	73.5% (58.1%–87.1%)
External test set II	0.789 (0.666–0.898)	72.5% (60.9%–82.6%)	84.0% (72.9%–93.6%)	42.1% (20.0%–66.7%)	81.6% (73.3%–89.1%)	79.2% (67.9%–89.8%)
External test set III	0.924 (0.852–0.980)	83.3% (75.0%–91.7%)	94.7% (87.7%–100.0%)	59.3% (41.4%–77.3%)	88.5% (82.1%–93.9%)	83.1% (73.4%–91.5%)
KNN-11						
Internal test set	0.853 (0.783–0.907)	79.8% (73.6%–85.4%)	86.9% (80.8%–93.0%)	64.3% (51.6%–77.1%)	85.5% (80.7%–89.8%)	84.1% (77.2%–90.2%)
External test set I	0.872 (0.758–0.953)	77.8% (64.4%–88.9%)	92.3% (79.4%–100.0%)	57.9% (35.3%–80.0%)	82.8% (72.0%–92.1%)	75.0% (59.5%–89.3%)
External test set II	0.780	71.0%	82.0%	42.1%	80.4%	78.8%

	(0.655–0.899)	(59.4%–82.6%)	(70.9%–91.8%)	(21.1%–66.7%)	(70.7%–87.9%)	(67.3%–89.4%)
External test set III	0.912	83.3%	93.0%	63.0%	88.3%	84.1%
	(0.844–0.973)	(75.0%–90.5%)	(85.7%–98.4%)	(44.4%–80.6%)	(82.0%–93.8%)	(74.6%–92.2%)

***AUC* area under the receiver operating characteristic curve, *KNN* K-nearest-neighbors**

Supplementary Table S9 Performance of KNN-based combined models using different K values with a cutoff value based on the best Youden index

Model	AUC	Accuracy	Sensitivity	Specificity	F1 Score	Precision
KNN-7						
Internal test set	0.849	82.0%	86.9%	71.4%	86.9%	86.9%
	(0.788–0.909)	(76.4%–87.6%)	(80.6%–92.2%)	(59.3%–83.0%)	(82.0%–90.9%)	(80.8%–92.5%)
External test set I	0.895	82.2%	92.3%	68.4%	85.7%	80.0%
	(0.789–0.965)	(71.1%–93.3%)	(81.8%–100.0%)	(45.0%–88.9%)	(74.5%–94.3%)	(65.7%–93.1%)
External test set II	0.796	75.4%	82.0%	57.9%	82.8%	83.7%
	(0.679–0.891)	(65.2%–85.5%)	(71.1%–91.5%)	(35.0%–78.9%)	(74.0%–89.9%)	(73.5%–93.6%)
External test set III	0.928	85.7%	91.2%	74.1%	89.7%	88.1%
	(0.858–0.981)	(78.5%–92.9%)	(83.1%–98.2%)	(56.0%–90.9%)	(83.2%–94.5%)	(79.7%–95.5%)
KNN-9						
Internal test set	0.851	81.5%	84.4%	75.0%	86.2%	88.0%
	(0.788–0.909)	(75.8%–86.5%)	(78.2%–90.2%)	(63.3%–86.5%)	(81.5%–90.5%)	(82.0%–93.7%)
External test set I	0.878	80.0%	92.3%	63.2%	84.2%	77.4%
	(0.767–0.959)	(66.7%–91.1%)	(80.0%–100.0%)	(38.9%–84.6%)	(72.7%–93.1%)	(63.0%–91.2%)
External test set II	0.789	72.5%	80.0%	52.6%	80.8%	81.6%
	(0.659–0.897)	(62.3%–82.6%)	(69.2%–90.0%)	(29.4%–73.7%)	(71.4%–88.9%)	(70.6%–91.5%)
External test set III	0.924	85.7%	91.2%	74.1%	89.7%	88.1%
	(0.854–0.978)	(78.6%–92.9%)	(83.6%–98.2%)	(56.2%–90.0%)	(83.3%–94.9%)	(78.8%–96.2%)
KNN-11						
Internal test set	0.853	80.3%	82.8%	75.0%	85.2%	87.8%
	(0.792–0.910)	(74.7%–86.0%)	(75.4%–89.3%)	(62.3%–86.0%)	(80.2%–89.8%)	(81.4%–93.7%)
External test set I	0.872	80.0%	92.3%	63.2%	84.2%	77.4%
	(0.768–0.953)	(68.9%–91.1%)	(81.5%–100.0%)	(40.0%–84.2%)	(73.5%–93.1%)	(62.1%–91.7%)
External test set II	0.780	73.9%	80.0%	57.9%	81.6%	83.3%

	(0.658–0.895)	(63.8%–84.1%)	(68.1%–90.9%)	(34.7%–81.0%)	(71.9%–88.9%)	(72.1%–93.5%)
External test set III	0.912	86.9%	89.5%	81.5%	90.3%	91.1%
	(0.841–0.966)	(79.8%–94.0%)	(81.4%–96.6%)	(66.7%–95.5%)	(83.9%–95.6%)	(83.3%–98.1%)

***AUC* area under the receiver operating characteristic curve, *KNN* K-nearest-neighbors**

Supplementary Table S10 Performance of KNN-based combined models in subgroup analysis

Subgroup	AUC	Accuracy	Sensitivity	Specificity	F1 Score	Precision
Age						
< 31 years	0.769	86.0%	99.3%	20.7%	92.2%	86.0%
	(0.661–0.857)	(80.7%–90.6%)	(97.8%–100.0%)	(6.7%–36.7%)	(88.8%–95.0%)	(80.7%–90.9%)
≥ 31 years	0.838	72.7%	73.5%	71.7%	74.8%	76.1%
	(0.780–0.885)	(65.9%–78.5%)	(66.0%–81.4%)	(62.2%–80.4%)	(67.4%–81.2%)	(67.9%–83.8%)
Sex						
Male	0.858	82.3%	90.5%	54.2%	88.8%	87.1%
	(0.793–0.908)	(77.7%–86.9%)	(86.4%–94.4%)	(42.4%–66.7%)	(85.4%–91.8%)	(82.7%–91.8%)
Female	0.802	70.7%	77.8%	64.5%	71.2%	65.6%
	(0.712–0.877)	(62.1%–78.4%)	(65.9%–88.0%)	(52.0%–76.1%)	(60.5%–80.0%)	(54.8%–76.6%)
Disease duration						
< 2 years	0.849	73.7%	85.7%	59.2%	78.0%	71.6%
	(0.790–0.903)	(67.1%–79.6%)	(78.8%–92.3%)	(48.0%–70.1%)	(71.3%–83.8%)	(64.0%–80.2%)
≥ 2 years	0.852	82.8%	89.0%	60.0%	89.0%	89.0%
	(0.778–0.909)	(77.5%–87.6%)	(83.9%–93.5%)	(45.2%–74.6%)	(85.4%–92.4%)	(84.2%–93.7%)
HLA-B27 status						
(+)	0.570	93.5%	95.6%	33.3%	96.6%	97.7%
	(0.352–0.881)	(89.8%–96.8%)	(92.7%–98.3%)	(0.0%–80.0%)	(94.6%–98.4%)	(95.4%–99.4%)
(-)	0.722	68.1%	68.8%	67.6%	64.1%	60.0%
	(0.622–0.818)	(59.5%–76.7%)	(54.2%–81.0%)	(56.3%–77.8%)	(52.3%–73.6%)	(47.1%–72.2%)
Unknown	0.756	58.1%	70.4%	51.1%	55.1%	45.2%
	(0.637–0.859)	(47.3%–68.9%)	(52.2%–87.0%)	(36.4%–66.7%)	(40.0%–67.6%)	(28.9%–59.6%)

***AUC* area under the receiver operating characteristic curve, *KNN* K-nearest-neighbors**

Supplementary Table S11 Performance of unrefined and refined models on the prospective validation set

Model	AUC	*p*	Accuracy	Sensitivity	Specificity	F1 Score	Precision
MRI-based model		0.651					
Unrefined	0.797 (0.706–0.879)		77.2% (70.3%–83.4%)	78.9% (70.8%–86.8%)	72.2% (57.6%–86.8%)	83.9% (78.5%–88.8%)	89.6% (83.3%–95.6%)
Refined	0.812 (0.732–0.888)		74.5% (66.9%–81.4%)	77.1% (68.5%–84.3%)	66.7% (51.2%–81.6%)	82.0% (75.9%–87.4%)	87.5% (80.0%–93.6%)
KNN-11-based combined model		0.501					
Unrefined	0.832 (0.753–0.894)		71.7% (64.8%–78.6%)	70.6% (61.9%–78.6%)	75.0% (59.3%–89.7%)	79.0% (72.1%–84.7%)	89.5% (82.9%–95.6%)
Refined	0.840 (0.767–0.900)		72.4% (64.8%–79.3%)	70.6% (61.8%–78.8%)	77.8% (63.3%–90.3%)	79.4% (73.2%–85.3%)	90.6% (84.6%–96.5%)

Delong's test was used to compare the AUCs of the unrefined and refined models

AUC **area under the receiver operating characteristic curve,** ***KNN*** **K-nearest-neighbors**

Supplementary Figures

Supplementary Fig. S1 The ASAS classification criteria for axial spondyloarthritis [3]

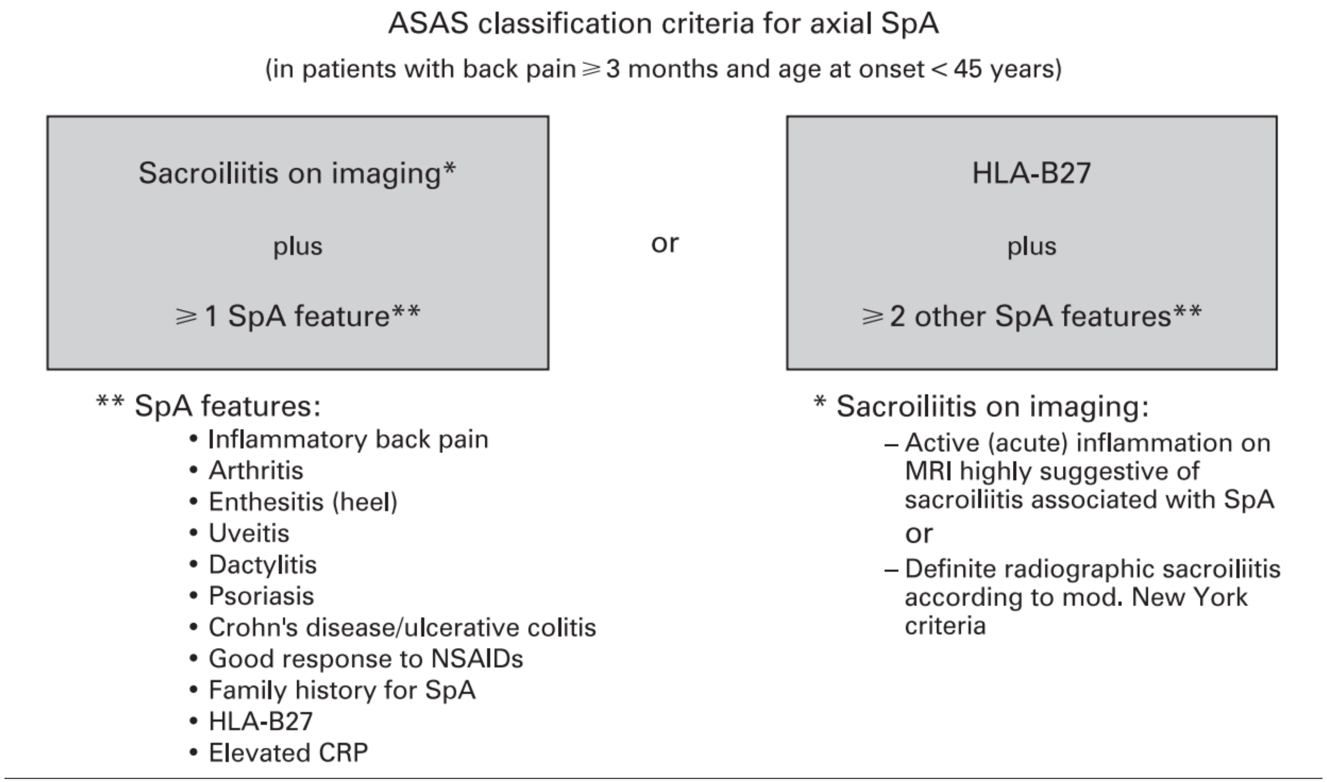

ASAS, Assessment of SpondyloArthritis International Society; SpA, spondyloarthritis; CRP, C-reactive protein; HLA-B27, human leukocyte antigen-B27; NSAID, non-steroidal anti-inflammatory drug

Supplementary Fig. S2 Proportion of patients with HLA-B27 status

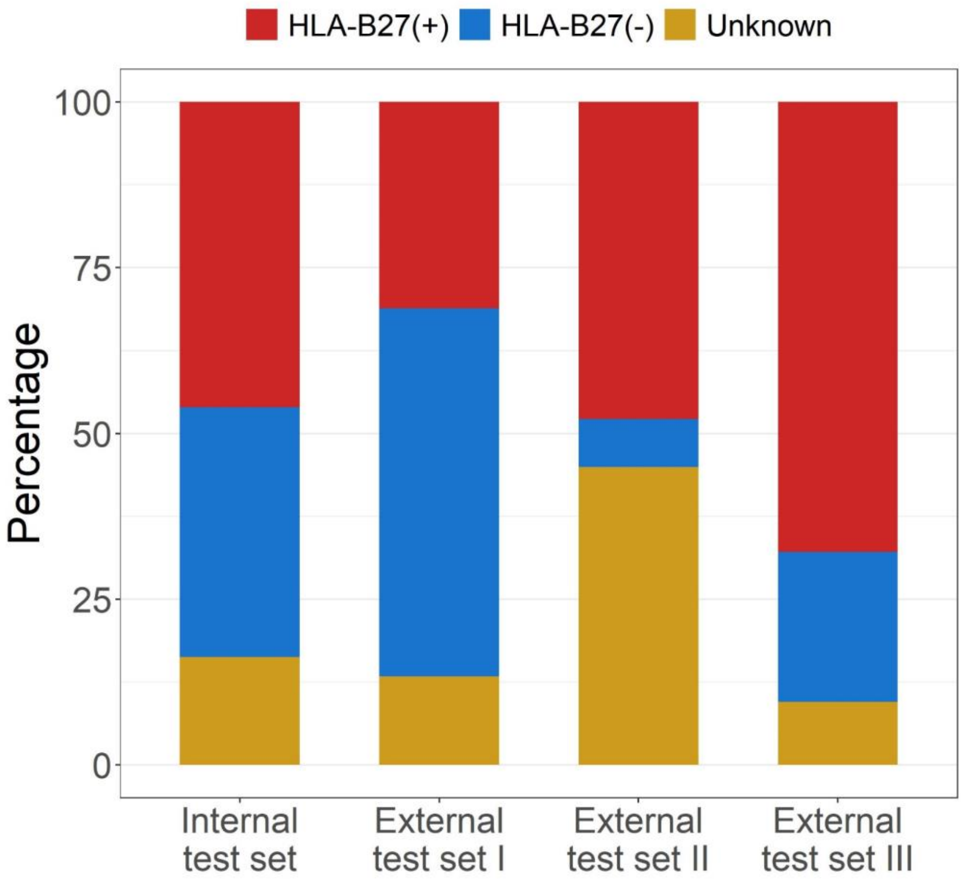

HLA, human leukocyte antigen

Supplementary Fig. S3 Density plots of original clinical data (black lines) and imputed clinical data (red lines)

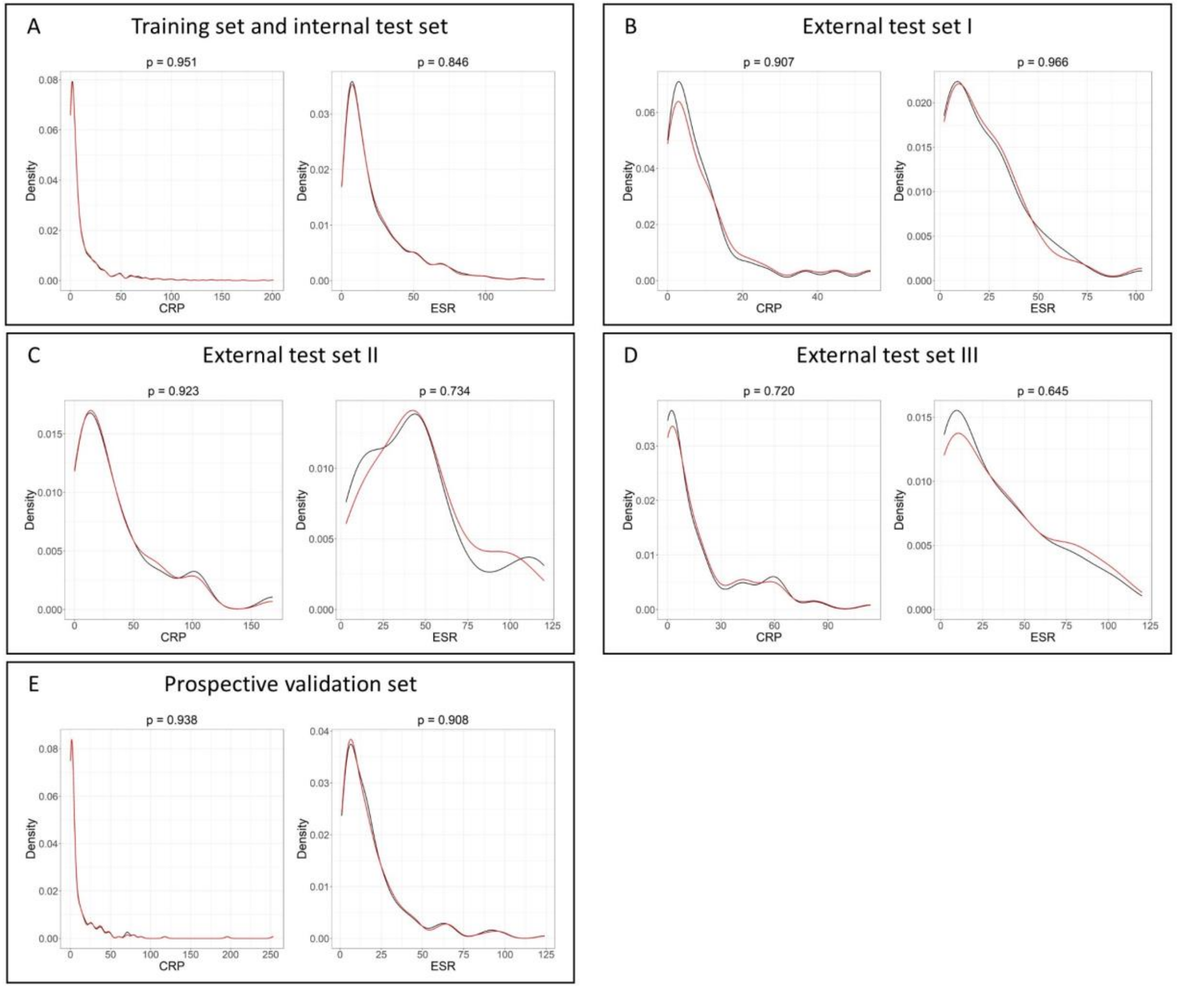

ESR, erythrocyte sedimentation rate; CRP, C-reactive protein

Supplementary Fig. S4 Representative cases incorrectly classified by the combined model

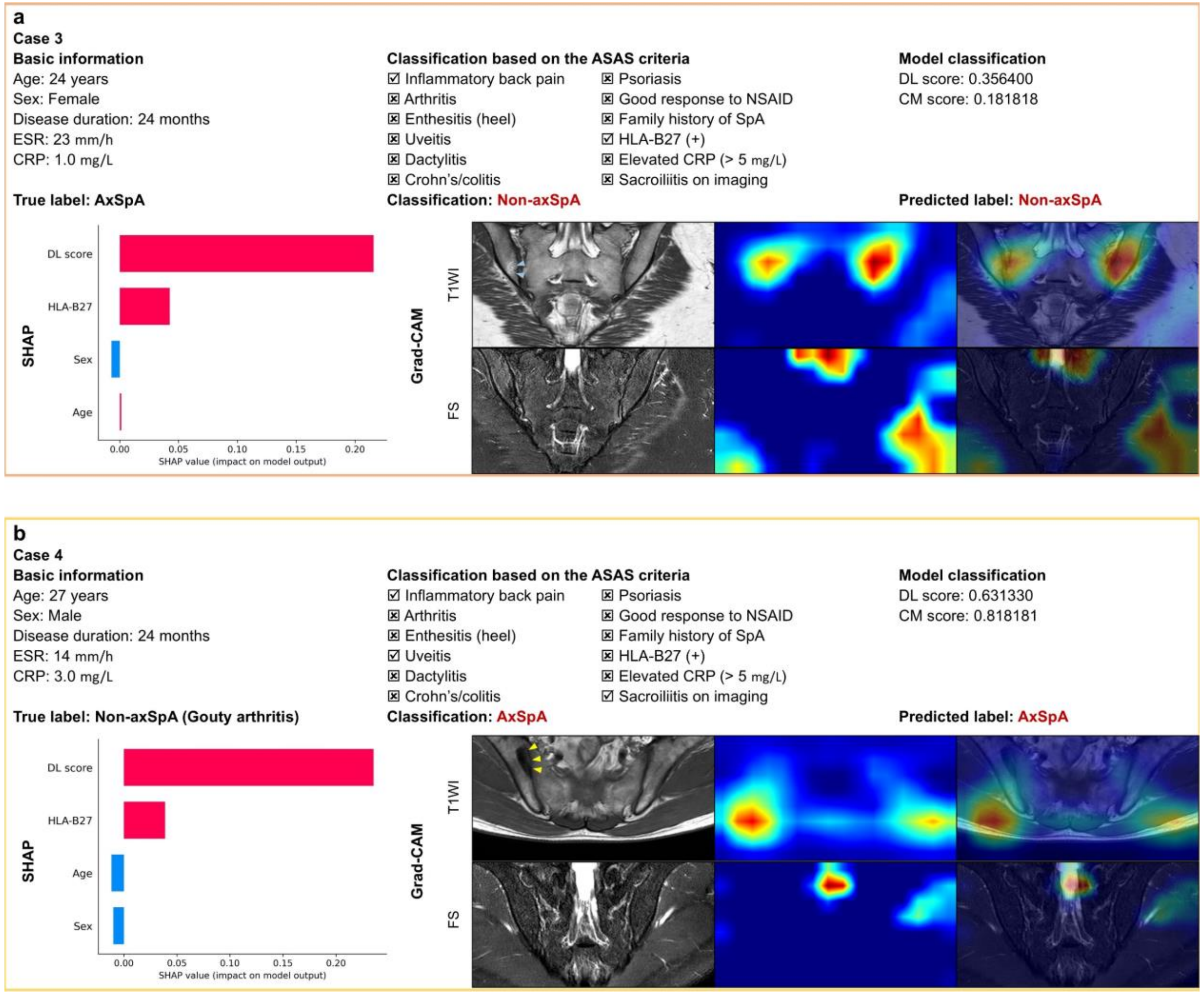

SHAP values are presented to indicate the contributions of various variables, with red highlighting potential risk factors for axSpA and blue denoting protective features. MRI heatmaps were generated using Grad-CAM, with red areas representing highly activated regions. The blue arrowheads indicate fat metaplasia. The yellow arrowheads indicate osteosclerosis. Text in blue denotes correct classifications, while text in red denotes incorrect classifications. ASAS, the Assessment of SpondyloArthritis International Society; axSpA, axial spondyloarthritis; CRP, C-reactive protein; ESR, erythrocyte sedimentation rate; HLA, human leukocyte antigen; DL, deep learning; CM, combined model; T1WI, T1-weighted imaging; FS, fluid-sensitive fat suppression; Grad-CAM, gradient-weighted class activation mapping; SHAP, Shapley additive explanation

Supplementary Fig. S5 Training dynamics across five-fold cross-validation

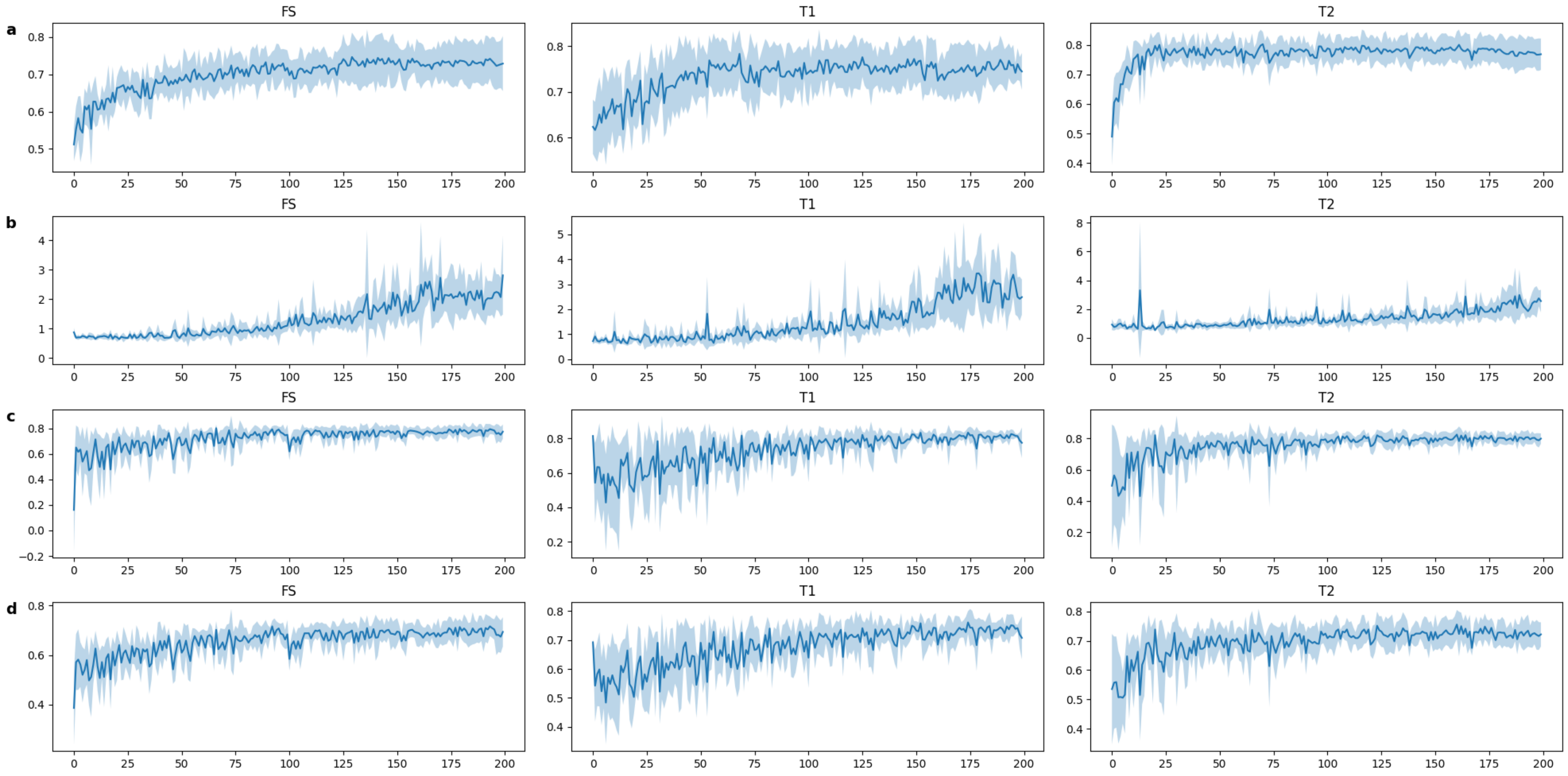

(a) Validation AUC curves across epochs demonstrate steady improvement, with peak convergence observed around 100–150 epochs. (b) Validation loss curves exhibit a slight upward trend following an initial decline, indicating potential overfitting beyond certain epochs. (c) Validation F1 score curves

show stable improvements in tandem with AUC. (d) Validation accuracy curves align with AUC trends, confirming consistent training behavior across folds. These plots illustrate the training stability and support the model selection strategy, where the optimal epoch is determined based on the highest validation AUC for each fold. AUC, area under the receiver operating characteristic curve

Supplementary Fig. S6 Effect of learning rate on model performance metrics across 200 epochs

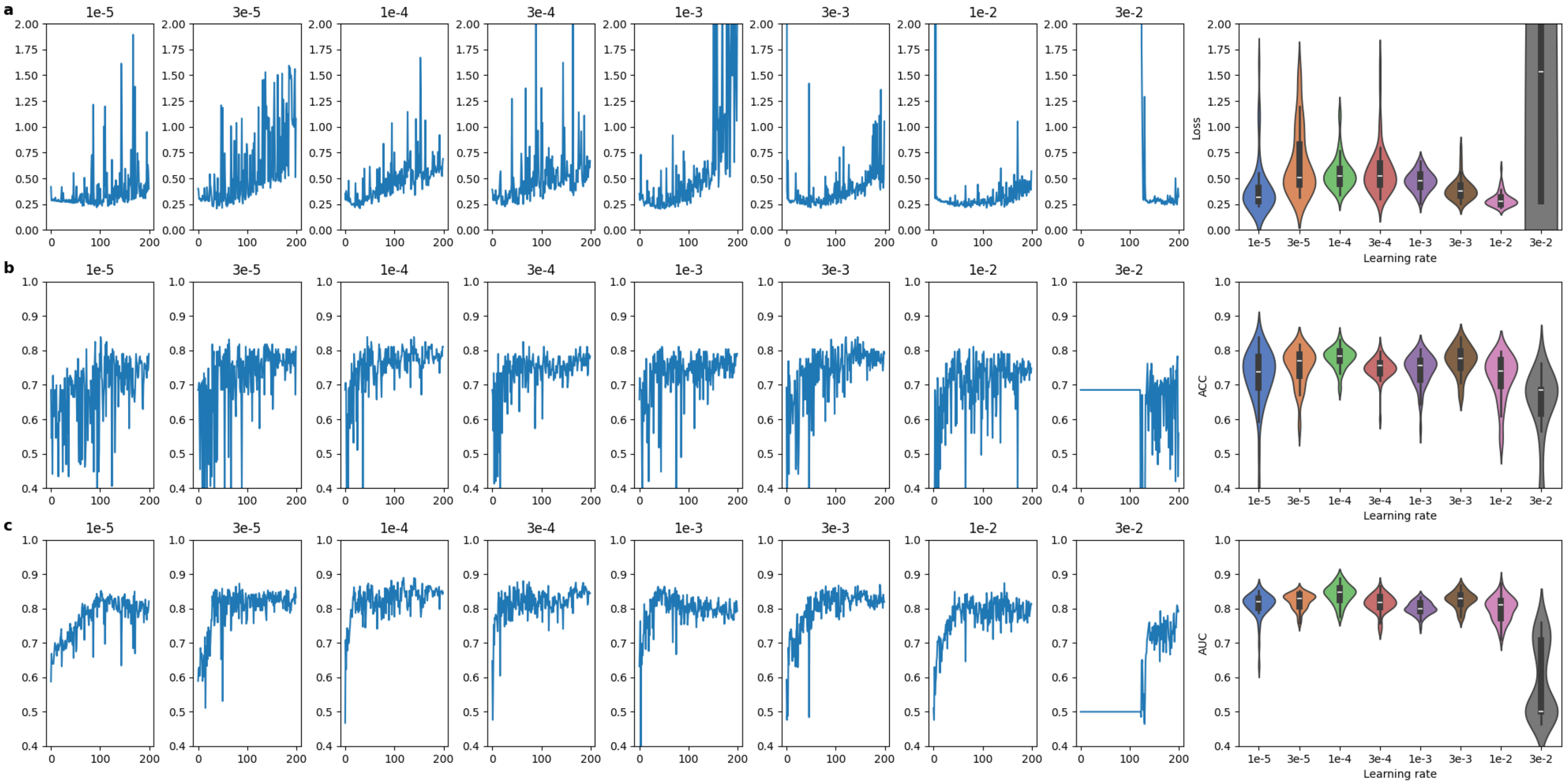

The effect of learning rate on model performance is systematically evaluated using grid search experiments. Rows (a), (b), and (c) represent the metrics of cross-entropy loss, ACC, and AUC, respectively. Each row features subplots displaying training curves for learning rates ranging from 1 × 10^{-5} to 3 ×

10^{-2}, increasing in a quasi-exponential manner over 200 epochs. The final subplots in each row illustrate the distribution of corresponding metrics across different learning rates using violin plots. The results indicate that smaller learning rates (e.g., 3×10^{-5} to 1×10^{-3}) facilitate more stable training and yield better performance, while excessively large learning rates (e.g., 3×10^{-2}) lead to unstable optimization and poorer results. ACC, accuracy; AUC, area under the receiver operating characteristic curve

References

1 van der Linden S, Valkenburg HA, Cats A (1984) Evaluation of diagnostic criteria for ankylosing spondylitis. A proposal for modification of the New York criteria. Arthritis and rheumatism 27:361-368

2 Maksymowych WP, Lambert RG, Østergaard M et al (2019) MRI lesions in the sacroiliac joints of patients with spondyloarthritis: an update of definitions and validation by the ASAS MRI working group. Annals of the Rheumatic Diseases 78:1550-1558

3 Rudwaleit M, van der Heijde D, Landewé R et al (2009) The development of Assessment of SpondyloArthritis international Society classification criteria for axial spondyloarthritis (part II): validation and final selection. Annals of the Rheumatic Diseases 68:777-783

4 Tustison NJ, Avants BB, Cook PA et al (2010) N4ITK: Improved N3 Bias Correction. IEEE Transactions on Medical Imaging 29:1310-1320

5 Messaoudi H, Belaid A, Salem DB, Conze P-H (2023) Cross-dimensional transfer learning in medical image segmentation with deep learning. Medical image analysis 88:102868

6 Zhang Y, Huang S-C, Zhou Z, Lungren MP, Yeung S (2022) Adapting Pre-trained Vision Transformers from 2D to 3D through Weight Inflation Improves Medical Image Segmentation. In: Antonio P, Monica A, Shalmali J et al, (eds) Proceedings of the 2nd Machine Learning for Health symposium. PMLR, Proceedings of Machine Learning Research, pp 391--404

7 Chen S, Ma K, Zheng Y (2019) Med3d: Transfer learning for 3d medical image analysis. arXiv preprint arXiv:190400625

8 Loshchilov I (2017) Decoupled weight decay regularization. arXiv preprint arXiv:171105101

9 Isensee F, Jaeger PF, Kohl SAA, Petersen J, Maier-Hein KH (2021) nnU-Net: a self-configuring method for deep learning-based biomedical image segmentation. Nature Methods 18:203-211

10 Selvaraju RR, Cogswell M, Das A, Vedantam R, Parikh D, Batra D (2017) Grad-cam: Visual explanations from deep networks via gradient-based localizationProceedings of the IEEE international conference on computer vision, pp 618-626

11 Lundberg SM, Lee S-I (2017) A unified approach to interpreting model predictionsAdvances in neural information processing systems,

12 Sieper J, van der Heijde D, Landewé R et al (2009) New criteria for inflammatory back pain in patients with chronic back pain: a real patient exercise by experts from the Assessment of SpondyloArthritis international Society (ASAS). Annals of the rheumatic diseases 68:784-788

STARD Checklist

Section & Topic	No	Item	Reported on page #
**TITLE OR ABSTRACT**			
	**1**	Identification as a study of diagnostic accuracy using at least one measure of accuracy (such as sensitivity, specificity, predictive values, or AUC)	Page 1
**ABSTRACT**			
	**2**	Structured summary of study design, methods, results, and conclusions (for specific guidance, see STARD for Abstracts)	Page 1
**INTRODUCTION**			
	**3**	Scientific and clinical background, including the intended use and clinical role of the index test	Pages 2–3
	**4**	Study objectives and hypotheses	Page 3
**METHODS**			
Study design	**5**	Whether data collection was planned before the index test and reference standard were performed (prospective study) or after (retrospective study)	Pages 3–4
Participants	**6**	Eligibility criteria	Supplementary Appendix S1
	**7**	On what basis potentially eligible participants were identified (such as symptoms, results from previous tests, inclusion in registry)	Page 3
	**8**	Where and when potentially eligible participants were identified (setting, location and dates)	Page 3
	**9**	Whether participants formed a consecutive, random or convenience series	Page 3
Test methods	**10a**	Index test, in sufficient detail to allow replication	Pages 4–5
	**10b**	Reference standard, in sufficient detail to allow replication	Supplementary Appendix S2
	**11**	Rationale for choosing the reference standard (if alternatives exist)	Supplementary Appendix S2
	**12a**	Definition of and rationale for test positivity cut-offs or result categories of the index test, distinguishing pre-specified from exploratory	Pages 7–8
	**12b**	Definition of and rationale for test positivity cut-offs or result categories of the reference standard, distinguishing pre-specified from exploratory	Supplementary Appendix S2
	**13a**	Whether clinical information and reference standard results were available	Supplementary Appendix S2

		to the performers/readers of the index test	
	**13b**	Whether clinical information and index test results were available to the assessors of the reference standard	Supplementary Appendix S2
Analysis	**14**	Methods for estimating or comparing measures of diagnostic accuracy	Page 6
	**15**	How indeterminate index test or reference standard results were handled	Page 4
	**16**	How missing data on the index test and reference standard were handled	Page 4
	**17**	Any analyses of variability in diagnostic accuracy, distinguishing pre-specified from exploratory	Page 6
	**18**	Intended sample size and how it was determined	Page 6
**RESULTS**			
Participants	**19**	Flow of participants, using a diagram	Page 3
	**20**	Baseline demographic and clinical characteristics of participants	Page 6
	**21a**	Distribution of severity of disease in those with the target condition	Not applicatble
	**21b**	Distribution of alternative diagnoses in those without the target condition	Supplementary Appendix S3
	**22**	Time interval and any clinical interventions between index test and reference standard	Not applicatble
Test results	**23**	Cross tabulation of the index test results (or their distribution) by the results of the reference standard	Pages 7–8; Table 2
	**24**	Estimates of diagnostic accuracy and their precision (such as 95% confidence intervals)	Pages 7–8; Table 2 and 3
	**25**	Any adverse events from performing the index test or the reference standard	Not applicatble
**DISCUSSION**			
	**26**	Study limitations, including sources of potential bias, statistical uncertainty, and generalisability	Page 11
	**27**	Implications for practice, including the intended use and clinical role of the index test	Pages 9–10
**OTHER INFORMATION**			
	**28**	Registration number and name of registry	Page 3
	**29**	Where the full study protocol can be accessed	Pages 4–5
	**30**	Sources of funding and other support; role of funders	Not applicatble

STARD 2015

AIM

STARD stands for “Standards for Reporting Diagnostic accuracy studies”. This list of items was developed to contribute to the completeness and transparency of reporting of diagnostic accuracy studies. Authors can use the list to write informative study reports. Editors and peer-reviewers can use it to evaluate whether the information has been included in manuscripts submitted for publication.

EXPLANATION

A **diagnostic accuracy study** evaluates the ability of one or more medical tests to correctly classify study participants as having a **target condition.** This can be a disease, a disease stage, response or benefit from therapy, or an event or condition in the future. A medical test can be an imaging procedure, a laboratory test, elements from history and physical examination, a combination of these, or any other method for collecting information about the current health status of a patient.

The test whose accuracy is evaluated is called **index test.** A study can evaluate the accuracy of one or more index tests. Evaluating the ability of a medical test to correctly classify patients is typically done by comparing the distribution of the index test results with those of the **reference standard**. The reference standard is the best available method for establishing the presence or absence of the target condition. An accuracy study can rely on one or more reference standards.

If test results are categorized as either positive or negative, the cross tabulation of the index test results against those of the reference standard can be used to estimate the **sensitivity** of the index test (the proportion of participants *with* the target condition who have a positive index test), and its **specificity** (the proportion *without* the target condition who have a negative index test). From this cross tabulation (sometimes referred to as the contingency or “2x2” table), several other accuracy statistics can be estimated, such as the positive and negative **predictive values** of the test. Confidence intervals around estimates of accuracy can then be calculated to quantify the statistical **precision** of the measurements.

If the index test results can take more than two values, categorization of test results as positive or negative requires a **test positivity cut-off**. When multiple such cut-offs can be defined, authors can report a receiver operating characteristic (ROC) curve which graphically represents the combination of sensitivity and specificity for each possible test positivity cut-off. The **area under the ROC curve** informs in a single numerical value about the overall diagnostic accuracy of the index test.

The **intended use** of a medical test can be diagnosis, screening, staging, monitoring, surveillance, prediction or prognosis. The **clinical role** of a test explains its position relative to existing tests in the clinical pathway. A replacement test, for example, replaces an existing test. A triage test is used before an existing test; an add-on test is used after an existing test.

Besides diagnostic accuracy, several other outcomes and statistics may be relevant in the evaluation of medical tests. Medical tests can also be used to classify patients for purposes other than diagnosis, such as staging or prognosis. The STARD list was not explicitly

CC BY SA

developed for these other outcomes, statistics, and study types, although most STARD items would still apply.

DEVELOPMENT

This STARD list was released in 2015. The 30 items were identified by an international expert group of methodologists, researchers, and editors. The guiding principle in the development of STARD was to select items that, when reported, would help readers to judge the potential for bias in the study, to appraise the applicability of the study findings and the validity of conclusions and recommendations. The list represents an update of the first version, which was published in 2003.

More information can be found on http://www.equator-network.org/reporting-guidelines/stard.

CC BY SA
